# Priorities for research to support local authority action on health and climate change: a study in England

**DOI:** 10.1186/s12889-023-16717-1

**Published:** 2023-10-10

**Authors:** Pete Lampard, Shainur Premji, Joy Adamson, Laura Bojke, Karen Glerum-Brooks, Su Golder, Hilary Graham, Dina Jankovic, Dagmar Zeuner

**Affiliations:** 1 University of York, York, UK; 2 London Borough of Merton, London, UK

**Keywords:** Mitigation, Adaptation, Evidence, Targeted reviews, Economic consequences, Public acceptability, Public involvement

## Abstract

**Background:**

Evidence is needed to support local action to reduce the adverse health impacts of climate change and maximise the health co-benefits of climate action. Focused on England, the study identifies priority areas for research to inform local decision making.

**Methods:**

Firstly, potential priority areas for research were identified from a brief review of UK policy documents, and feedback invited from public and policy stakeholders. This included a survey of Directors of Public Health (DsPH) in England, the local government officers responsible for public health. Secondly, rapid reviews of research evidence examined whether there was UK evidence relating to the priorities identified in the survey.

**Results:**

The brief policy review pointed to the importance of evidence in two broad areas: (i) community engagement in local level action on the health impacts of climate change and (ii) the economic (cost) implications of such action. The DsPH survey (*n* = 57) confirmed these priorities. With respect to community engagement, public understanding of climate change’s health impacts and the public acceptability of local climate actions were identified as key evidence gaps. With respect to economic implications, the gaps related to evidence on the health and non-health-related costs and benefits of climate action and the short, medium and longer-term budgetary implications of such action, particularly with respect to investments in the built environment. Across both areas, the need for evidence relating to impacts across income groups was highlighted, a point also emphasised by the public involvement panel. The rapid reviews confirmed these evidence gaps (relating to public understanding, public acceptability, economic evaluation and social inequalities). In addition, public and policy stakeholders pointed to other barriers to action, including financial pressures, noting that better evidence is insufficient to enable effective local action.

**Conclusions:**

There is limited evidence to inform health-centred local action on climate change. More evidence is required on public perspectives on, and the economic dimensions of, local climate action. Investment in locally focused research is urgently needed if local governments are to develop and implement evidence-based policies to protect public health from climate change and maximise the health co-benefits of local action.

**Supplementary Information:**

The online version contains supplementary material available at 10.1186/s12889-023-16717-1.

## Background

The mission of public health is to ensure the conditions in which people can live healthy lives [1, 2]. Driven by increasing greenhouse gas emissions and rising global temperatures, climate change is undermining these conditions [3–5]. In the UK, exposure to flooding [6] and heatwaves [7–9] has been identified as particular health risks. The UK National Risk Register highlights these climate-related exposures and places their human health impacts at the top of its list of adverse consequences [10]. In addition, it identifies air pollution as ‘the largest environmental risk to public health in the UK’ [10]. The health impacts of climate change differentially affect those at heightened risk of social disadvantage, including children, older people, poorer communities, minority communities and those with underlying health conditions [11, 12]. Lifetime risks of health-damaging exposures will increase across cohorts – so for children and for future generations compared with today’s adults, particularly if the upward trend in global temperatures is not halted [13].

Reducing emissions is recognised to bring important health benefits. For example, shifting from high-emitting travel modes to ones with lower carbon intensity (walking, cycling, electric vehicles) reduces population exposure to co-emitted air pollutants like fine particulate matter (PM_2.5_) and nitrogen dioxide (NO_2_) [14]. This, in turn, brings major health benefits, including avoided deaths from air pollution and improvements in health from increased active travel [15]. Compared to the longer-term and diffuse benefits of climate policies, these health co-benefits can be measured over relatively short time frames [16]; they can also differentially benefit vulnerable groups, including socially-disadvantaged groups and children [17].

The UK’s 2008 Climate Change Act [18], strengthened in 2019 [19], commits the UK to becoming a net zero society by 2050 – a commitment requiring a 68% reduction in carbon emissions by 2030 [20], for example through reducing emissions from road transport, buildings and waste management [21]. Recognition that mitigation alone will not protect the population from climate change has resulted in a greater emphasis on reducing vulnerability and moderating the adverse impacts of a changing climate (adaptation). The National Adaptation Programme seeks to address environmental risks to communities, including from flooding, air quality and high temperatures [22]. Again, adaptation measures – including local nature-based solutions - can have measurable health co-benefits [23, 24].

UK policies on climate change are emphasising the importance of action by local government [25–27]. Elected by their local populations, this tier of government has leverage over major drivers of greenhouse gas emissions, including energy use by road transport and in residential buildings. Via their responsibilities for transport planning, housing, leisure and environmental health, it is estimated that local government can influence around a third of emissions in their local areas [27]. Local government actions can also help to protect people’s health from changes in the climate that can no longer be prevented, for example, through flood risk management. Like local governments elsewhere [28], local authorities (LAs) in the UK are putting climate action plans in place. Over 83% of LAs have declared climate emergencies, with many making commitments on emissions reductions and on adapting to climate change [29, 30].

Local action is also integral to UK health policy. Health system governance varies across the devolved governments (Northern Ireland, Scotland, Wales), including public health governance [31, 32]. In England, in which 84% of the UK population live [33, 34], the UK government retains responsibility for health. England’s health system has undergone two decades of continuous change [32], the latest of which was formalised in the Health and Social Care Act (2022). This established Integrated Care Systems (ICSs), with two components: Integrated Care Boards (ICBs) and Integrated Care Partnerships (ICPs). ICSs are designed to improve the health of local populations through better collaboration between local National Health Service (NHS), LA organisations and the voluntary sector [35]. The Act requires each of the 42 ICBs to deliver their local Green Plan, assigning a Board-level lead for sustainability.

This shift towards ‘localism’, with its emphasis on local-level solutions, is occurring within the complex structures of local government.^1^ Directors of Public Health (DsPH) are the officers responsible for public health in the local government’s geographical area and ‘have a vital leadership role for system-wide efforts to secure better public health’ [36], often as part of a wider climate action plan [37, 38].

Against this policy backdrop, our study aims to identify research priorities to support action by LAs to address the health impacts of climate change [27, 39–41]. This includes economic evidence, where local decision makers can find it challenging to consider the case for investment or disinvestment in the context of interventions and programmes that have costs and outcomes falling on multiple sectors, including health, education and the wider economy. These multi-sector approaches can be complex to evaluate, particularly when costs and benefits occur further downstream [42, 43]. It should be noted that our focus is on identifying research priorities; it does not extend to reviewing findings of studies located through the study’s methods.

## Methods

**Fig. 1:**
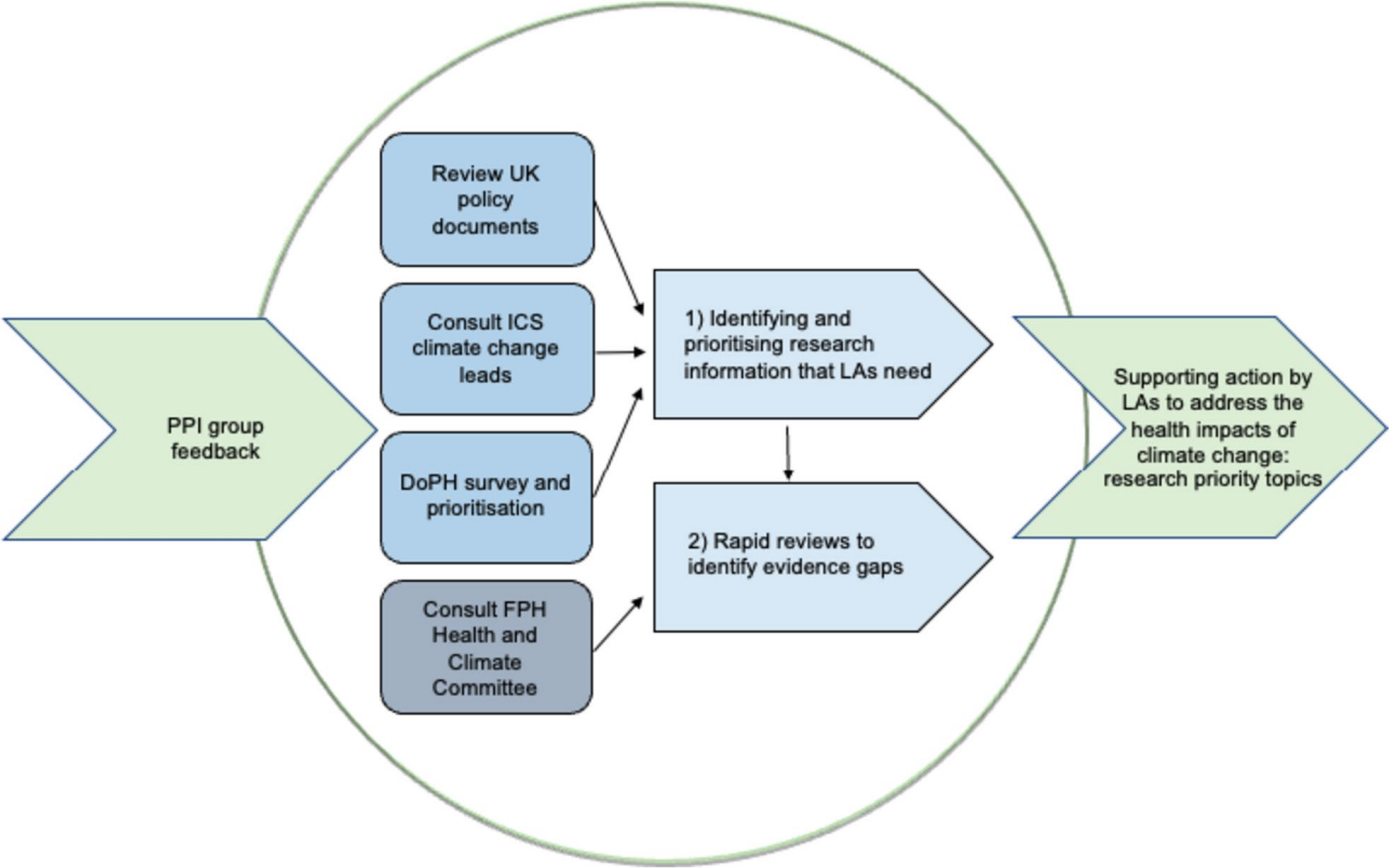
Study components

To identify key research priorities to support action by Local Authorities (LAs), we undertook two key activities: (1) identifying and prioritising the research information that LAs need and (2) conducting rapid reviews of research on these priorities to identify evidence gaps (Fig. 1). This work was undertaken as part of a 6 month study funded by the National Institute of Health Research (NIHR), the major funder of health research in England. The study was approved by the Research Governance Committee, Department of Health Sciences, University of York (ref: Re: HSRGC/2022/516/F: Local authority-level research priorities on climate change).

1. *Identifying and prioritising research information that LAs need.* This activity included a brief review of UK policy documents and advice from policy stakeholders and from members of the public (Fig. 1).

For the document review, we included all publicly available LA action plans (as of July 2022), accessed via *Climate Emergency UK* [44], together with reports from UK bodies responsible for advising the UK government on climate change and health. Documents were uploaded and searched using textual data analysis software in *R* (*Tabulizer* [45] and *Quanteda* [46]) for references to local level policies relevant to climate-related exposures (e.g. flooding, heat, air pollution), health and evidence gaps. The review was informed by public health and climate change frameworks derived from previous research [12, 47] and focused on research priorities and evidence gaps noted in the documents, together with broader references to health (Supplementary file 1). This brief review generated a set of potential research priority areas.

A draft of the priorities was distributed for feedback to an informal network of those engaged in sustainability leadership across the Integrated Care Systems (ICSs). Distributed via the group administrator to protect respondent anonymity, information on the profile of participants is not available. At the time of contact, Integrated Care Boards (ICBs) had only just become legal entities and not all sustainability leads had been appointed. Seven written responses were obtained.

Informed by policy document review and ICS sustainability stakeholders, an online survey of Directors of Public Health (DsPH) was conducted in August 2022. The survey was emailed to DsPH (*n* = 151) listed by Public Health England, a national agency with responsibility for protecting and improving public health,^2^ and publicised by the Association of Directors of Public Health (ADPH), the membership body of DsPH [48]. The survey invited DsPH to rank the research priority areas and to add further areas where they considered more evidence was needed (see Supplementary file 2). DsPH were asked to optionally record their LA, allowing us to explore whether priorities differed between rural and urban areas and between more and less deprived areas. Additionally, we examined whether LAs with climate action plans were overrepresented in the sample. Following the survey, a summary of findings was made available to DsPH via the ADPH and the project website [49]. To gain additional stakeholder input, a summary of survey findings was shared with the Climate and Health Committee of the Faculty of Public Health, a membership organisation representing UK public health professions [37, 50].

Additionally we spoke with members of the public, adhering to principles outlined in the UK standards for public involvement in research [51] and NIHR guidance for researchers [52]. The group, made up of both men (*n* = 2) and women (*n* = 3) from rural and urban areas with ages ranging from young adult to those in their seventies, included those at heightened vulnerability to climate-related risks to their health. Members of the group were personally approached and invited to contribute by the project’s public involvement manager. The project was introduced at their first meeting, and members discussed the impact of climate change on their lives and health, their LA’s role in making action possible and the barriers to climate action, including the potential value of research. The second meeting discussed the research priorities identified through the policy document review and the survey of DsPH. A summary of the project’s findings were shared with the group, and feedback was invited on the findings and on the public involvement process [53].

(2) *Conducting rapid reviews to establish whether there was UK research relating to the evidence that LAs need.* Rapid reviews systematically map the evidence on ‘urgent and emergent’ policy challenges [54, 55] to inform decision making and identify priorities for future research [55]. They streamline the processes used in traditional systematic reviews [56], for example, omitting appraisal of study quality. In line with Cochrane methodological guidance [54], the review topics were identified and refined with advice from key stakeholders (including DsPH and the public, as noted above).

The top four research priorities identified in the DsPH survey were selected for rapid review. We followed methods outlined by Cochrane [54], Arksey and O’Malley [57] and PRISMA reporting guidelines [58]. The protocols, including review questions, PICOS and inclusion/exclusion criteria, were registered on the UK’s Research Registry [59]. In brief, searches were conducted for studies published up to 1^st^ September 2022, in online bibliographic databases (MEDLINE, Embase, PsycINFO, HMIC and, additionally for the economic evaluation reviews, IDEAS and EconLit) using search terms relevant to the priority area; in addition, we conducted forward citation searching using snowballing for six iterations. Study designs eligible for inclusion were primary studies that included the UK general population. Studies were independently identified by two reviewers, including an information specialist, with a third reviewer check where there was uncertainty over inclusion. A standardised data extraction form was developed using Covidence [60] to summarise the studies, which were examined for evidence on social inequalities. Further information can be at the UK Research Registry [61–63].

## Results

### Identifying and prioritising research information that LAs need

The brief review of local national policy documents located few research priorities and evidence gaps specifically framed around climate action and people’s health. Instead, priorities were articulated in more general ways: around public engagement, communication and acceptability and around inequalities in vulnerability and impacts (see examples in Table 1). Evidence to inform economic decision making, including long- and short-term cost-benefit analyses, was more explicitly noted as a priority (Table 1). Further details are in Supplementary file 3.

**Table 1:** Examples of priority areas from the brief policy document review

***Public engagement and inequalities***
*There is currently a lack of understanding at a community level of how climate hazards may impact people and communities. (Bristol City Council, 2020)*
*Communication will certainly be key to ensuring that Dundee is resilient to climate change. (Dundee City Council, 2019)*
*Researching and evidencing the specific local physical, mental, and perceived barriers to active travel in different circumstances and in different parts of the district in order to more effectively target and support engagement, education, incentives and interventions (Somerset West and Taunton Council, 2020)*
*Climate change will exacerbate existing environmental inequalities, since some groups will be more affected by climate risks or have less capacity to prepare for them. We want to ensure no group is left behind by climate change in line with the government’s levelling up commitments… [We need] to better understand and integrate thinking on how we can reduce inequalities as a result of climate change. (Environment Agency, 2021)*
***Financial strategy and decision making***
*There is a gap in understanding of the quantified economic impacts of climate hazards at a local level. We recommend quantification of economic impacts of climate hazards in Bristol is undertaken to help build a business case for action… This would aid understanding of the economic viability of climate adaptation and assist in stimulating funding for climate adaptation measures (Bristol City Council, 2020)*
*Although adaptation and mitigation action may be expensive initially, if whole life costs are considered, often such measures tend to be cheaper than business as usual in the long term. Short term costs are often worth the savings across multiple departments and levels in the long term. (Sefton Metropolitan Borough Council, 2019)*
*Many measures will have a financial return on investment, but many may not. However, many will have wider health and economic benefits which can be realised. As further work is done to draw up detailed implementation plans for our buildings, vehicles, and energy infrastructure, we will need to develop a detailed financial strategy (Dorset Council, 2022)*

The ICS sustainability stakeholders were in broad agreement with the priorities, giving particular emphasis to social inequalities in climate-related exposures and health impacts. However, there was a strongly expressed view that working with communities to develop and implement climate resilience plans (‘to make changes happen on the ground’) was a greater priority than more research to fill evidence gaps.

The stakeholder survey was completed by 57 DsPH (38% response rate) and one non-DPH. The large majority of DsPH (51; 90%) recorded the LA they represented. This enabled sample representativeness to be assessed by population density (rural/urban areas) and by area deprivation. For the latter analyses, we used the Index of Multiple Deprivation (IMD), a measure of relative deprivation at small area level.^3^ The LAs were representative of LAs in England with respect to deprivation (mean IMD 23.0 compared to 21.7 for England as whole) but were more populated (3,082 people per square km compared to 432 for England). IMD and population density were not correlated (Pearson correlation coefficient 0.21). The proportion of LAs with a climate action plan (77%) was comparable to the proportion nationally (74%). For further details of sample representativeness, see Supplementary file 4.

The research priorities selected by the survey participants are summarised in Table 2. With respect to community engagement, the public acceptability of local actions (85%) and public understanding of climate change and its impacts on people’s health (74%) were most frequently selected. With respect to understanding the economic (cost) implications of local actions, the most frequently selected areas were evidence on the health and non-health-related costs and benefits of investing in climate change mitigation and adaptation activities (76%) and information on the short, medium, and long-term budgetary implications of climate change mitigation and adaptation activities (69%). Economic evidence relating to investments in the built environment (including building design and healthy homes schemes) was seen as a greater priority than evidence on costs and benefits of actions relating to food (promoting healthier diets and sustainability of food supply) and physical activity (active travel infrastructure and active lifestyles). Across both community engagement and economic evaluation, evidence relating to different income groups was identified as a particular priority (selected by 66% of DsPH for both areas).

**Table 2:** Top three research priorities selected for each survey question

**Survey question**	**Top three research priorities identified in response to each question**
***Public engagement in local action***	
Areas where more evidence is needed by your local authority on ways to engage the public in local action to mitigate and adapt to the health impacts of climate change	1. The public acceptability of local actions (e.g. low traffic neighbourhoods) (85%) 2. Public understandings of climate change and its impacts on people’s health (74%) 3. Best practice in engaging with local businesses (48%)
Groups or communities where more evidence is needed on effective ways to engage the public in local level actions to mitigate and adapt to the health impacts of climate change on health	1. Different income groups (e.g. richer and poorer households) (65%) 2. Communities facing barriers to decent housing and local services (44%) 3. Communities from different ethnic and cultural backgrounds (41%)
***Economic (cost) implications of local actions***	
Areas where more evidence is needed to understand the economic (cost) implications of actions to mitigate and adapt to the health impacts of climate change	1. Evidence on the health and non-health-related costs and benefits of investing in climate change mitigation and adaptation activities (76%) 2. Information on the short, medium, and long-term budgetary implications of climate change mitigation and adaptation activities (69%) 3. Best practice evidence on policies to financially incentivise local businesses to adopt climate change mitigation and adaptation activities (48%)
Specific sectors where more evidence is needed to understand the economic (cost) implications of actions to mitigate and adapt to the health impacts of climate change	1. Built environment, building design, healthy homes schemes (60%) 2. Healthier diets and sustainability of food supply (48%) 3. Active travel infrastructure and active lifestyles (38%)
Groups or communities where more evidence is needed to understand the economic (cost) implications of actions to mitigate and adapt to the health impacts of climate change	1. Different income groups (e.g. richer and poorer households) (66%) 2. Communities facing barriers to decent housing and local services (50%) 3. All communities (45%)

Most (32; 56%) DsPH added textual comments. Many comments reiterated the importance of evidence in the priority areas already identified. However, others pointed to wider barriers to action. These related to climate change governance, including tensions and misalignments between national and local agenda, and, at local level, a lack of engagement in climate actions and its economic co-benefits among elected members of LAs, service commissioners and community leaders. Budget constraints and shortfalls were also highlighted as barriers, with the result that, as one participant observed, ‘asking for more evidence may be a delaying tactic’. Evidence on implementation (approaches that had worked well/less well) was also noted to be a priority.

Feedback on the DsPH survey findings was provided by the FPH Climate and Health Committee. In line with textual comments from DsPH, the Committee noted an ‘implementation gap’ around putting evidence into practice at local level. The FPH Committee also highlighted the need for evidence on how to change the behaviour of higher income groups, and noted a ‘disconnect’ between local and national government, including the lack of resources at local level to support climate action.

At their meetings, the public involvement panel spoke of how extreme weather was already affecting their health, leisure, social life, and feeling of wellbeing. They spoke of actions they were taking to try to minimize their impact on the environment. They discussed how they would like to do more, but a sustainable lifestyle can be quite expensive and that LAs were struggling to make some initiatives (for example, emission reduction zone charges) fair to everyone. Panel members expressed the view that, rather than research evidence, LA’s lack of money and being out of touch were the biggest barriers to implementing local change actions. They highlighted the need to engage with people ‘at ground level’. Actions taken during Covid, when council staff went into the communities, were mentioned as an example of good practice. Contributors also felt strongly that research on climate change and public health should focus on finding ways to tackle social inequalities and promote fairness within their communities.

In their second meeting, our public involvement panel spoke about the priority areas from the DsPH survey. Their feedback is summarised in Table 3 below.

**Table 3:** Feedback on the priority areas from the public involvement panel

***Public understanding of the health impacts of climate change***
Some of our PPI contributors said their health was already suffering from the effects of climate change. One member had a lung condition that is severely affected by damp weather, when they have to stay on their breathing machine during the day. Contributors said they find it very important that people understand what is happening in their local area in terms of impact and exposure - not just in the future but also right now. They believe that understanding the link between climate change and health may make climate change more relevant to people’s own lives and motivate people to live more sustainably. Knowing where to find help to live more sustainably is also considered important, as is knowing how to keep the damp out of your house, or to keep it cool during summer. Our school-aged contributor pointed out that the link between climate change and health is not covered at GCSE level, even though climate change is a considerable part of the curriculum.
***Public acceptability of local climate actions***
Some contributors feel that radical climate action groups are putting people off and making it harder to talk about climate change. They said it would be helpful to focus on health rather than the world at large. Others want LAs to do what is necessary and not always worry about popularity. They also feel that encouragement to live sustainably should come from showing what other people were doing and from showing local progress. Another contributor noted that a lot of decisions on climate change seem to come from central government, and that we should take more responsibility for what we are causing elsewhere in the world.
***Budgetary and economic implications of climate action***
PPI contributors advocated a holistic approach. They feel that robust communities can counterbalance health threats and help people live more sustainably. In this context, one contributor pointed out that small actions can have considerable impact: repairing bus shelters quickly, public seating in town centres, public herb gardens. Contributors spoke of the importance of finding a way to harness the power within the communities themselves. They believe that LAs should be monitoring the effects of climate action for the benefit of the community. One said: ‘They should help us, engage with us, collect the right data and make it accessible. Develop the evidence, we need strong evidence!’

### Rapid reviews of UK evidence on the research priorities to identify evidence gaps

The rapid reviews pointed to a very limited UK evidence base on the areas identified as gaps in the DsPH survey. This was particularly true of the two economic impact priorities highlighted in Table 2, relating to the costs and benefits of investing in climate change mitigation and adaptation activities and their short, medium and long-term budgetary implications. The two areas were therefore combined into a single rapid review. Further details of the findings of the three reviews, including EviAtlas [64] spatial maps of the study sites, are in Supplementary file 5.

Twenty seven studies (reported in 30 publications) were identified relating to public understanding of the health impacts of climate change in the UK. Only six studies explored the perceptions of the health impacts of climate change (rather than a climate change-related exposure such as flooding or heatwaves), and only one of these had perceptions of the health impacts of climate change as its primary focus. With respect to social inequalities, only a minority of studies reported patterns by gender (7 of 27). Similarly, only a minority (*n* = 5) reported findings by socioeconomic group (income/financial strain, educational attainment, employment status, housing) and by age or age cohort. Only one study reported on differences by ethnic group.

The review located a larger UK evidence base on the public acceptability of local climate actions: 110 research studies (reported in 117 publications). The large majority of studies (*n* = 56) related to the public acceptability of actions addressing energy generation, including the siting of renewable energy development (for example, we located 20 studies on wind power development); in contrast, there was only one study each on the acceptability of reduced street lighting and energy efficiency in the home. None of this group of studies report findings by socio-economic group or ethnic group. The other major group of studies (*n* = 33) related to the public acceptability of recycling schemes. Only a minority (9 studies) reported findings by social position.

Across the two economics topics, there was very little UK evidence (five separate studies in five publications) which attempted to quantify either the cost-effectiveness of climate actions or their budgetary implications. There was no evidence which measured short, medium, or long-term impacts on LAs, or on other local level UK decision-making entities. This lack of evidence is surprising given the likely significant budgetary impacts of some climate actions that could be implemented by LAs, for example switching to electric fleets for school transport. The five economics studies attempted to quantify either the cost-effectiveness or the cost–benefit of a climate action compared to a ‘do nothing’ option. The interventions related to waste management, ecology restoration, home energy, air pollution and transport. Each study used data from local changes/initiatives, two of which were London based, but all attempted to generalize findings across the UK narratively and not quantitatively. All but one study, relating to waste management, included a limited time horizon to quantify comparative costs and outcomes, likely resulting in an inaccurate understanding of the cost-effectiveness of the various climate actions. All but one of the studies – which used a societal perspective to evaluate two competing waste management systems [65] - considered a more limited perspective for costs and outcomes, for example, city level or health system perspective.

## Discussion

This two-staged study aimed to identify priorities for research to inform local action on climate change and health in England. It began by mapping and prioritising research needs through a brief review of policy documents and stakeholder consultations, including a survey of DsPH, the public health leaders in LAs. It then undertook rapid reviews of evidence in the identified priority areas: public understandings of health and climate change, public acceptability of climate action and the economic implications of such action for effective local action. The reviews pointed to a dearth of UK evidence in all these areas.

Very few studies explored public perceptions of the climate change-health nexus, with the majority focusing on climate-related exposures as standalone issues, often with little or no mention of climate change to the study participants. This is a noteworthy gap, given evidence that a health framing of climate change can promote public engagement in climate action [66–68] (a point also noted by our public involvement panel, see Table 3).

There was a larger body of evidence on the public acceptability of local actions. However, studies focused on a limited set of actions: energy generation, particularly the siting of potential renewable energy investments, and recycling schemes. The review located very few studies relating to a broader range of climate actions, for example around sustainable food and adaptation actions (e.g. flood risk management).

While evidence in these key areas of public understanding and acceptability was limited, the major gap related to evidence on the economic implications of climate actions. The review located only a small group of studies. Further, while the DsPH survey highlighted the need for evidence of the economic cost implications of climate actions related to the built environment (Table 2), only one study (of home energy) addressed this priority [69]. The importance of these gaps is underlined when set in a co-benefits perspective: accounting for the health co-benefits of effective mitigation and adaptation action can substantially reduce economic costs, including in the near-term [15, 24].

The advice from the public involvement group underlined the importance of social inequalities. The group made clear that individuals may wish, but be unable to afford, to live more sustainably, and fairness needed to be a guiding principle of local action on health and climate change. A lack of engagement in inequalities and social justice was evident across all priority areas (public understanding, public acceptability, economic evaluation). Thus there were gaps around whether there were differences in public perceptions of climate-related health impacts and in the acceptability of local climate actions between social groups. No study in the economic review provided evidence relating to the equity implications of local actions - for example, the distribution of costs and benefits across richer and poorer groups.

Some limitations of our study should be noted. Firstly, the constituent parts of the study (Fig. 1) were truncated by its short timeframe. Each element - the policy review, public involvement, stakeholder consultation and the evidence reviews - was reduced in scope and depth to enable the project’s completion within 6 months. Nonetheless, all elements were completed and their findings integrated. The rapid reviews were informed by best practice guidelines [54]; this included feedback on scope from public and policy stakeholders, the publication of the review strategies [61–63], restriction of the publication language to English, searches of major databases but with specialised database searches where relevant (economic evaluation), forward citation searching and the involvement of two reviewers, including an information specialist. As a rapid review, appraisal of study quality was not undertaken; it is therefore probable that the pool of high quality studies is smaller than the already limited number of studies identified by our review. Additionally, the review did not include an evidence synthesis, a stage that would have been likely to underline the paucity of evidence to inform local action on health and climate change.

Secondly, the study was commissioned to inform research in England [70] and did not focus on UK countries where there may be a wider and potentially-relevant literature. Devolution in the UK - with Northern Ireland, Scotland and Wales having their own elected governments and devolved responsibilities - has resulted in policy divergence in areas such as health and environment [31, 32]. However, policies in England have remained under the UK government, implemented through the multi-level structures of local government. In this complex policy environment, the components of our study have involved different spatial scales. The policy document review drew on UK-wide policy documents, including local climate action plans. Similarly, the rapid reviews searched for UK evidence. However, to ensure the study fulfilled its remit of informing future research to support local action health and climate change in England, the prioritisation exercise focused on stakeholders in England. It involved members of the public and local public health leaders (DsPH) in England. We acknowledge that a longer study could have investigated divergences in local climate action plans between England and the devolved governments, and included a research prioritisation exercise among local government public health leaders in Northern Ireland, Scotland and Wales.

Nonetheless, the study design (Fig. 1) generated a clear set of research gaps, relating to the need for more evidence on public understanding, public acceptability and economic evaluation. Additionally, the study found evidence of a counternarrative, one that disputed the need for more research. In line with a recent UK study of health-focused urban decision making [71], some policy stakeholders pointed to the need to address governance barriers, both between national and local government and between departments within local authorities (e.g. environment, housing, transport and health), and public stakeholders pointed to barriers to action resulting from LA relationships with the public.

Framing these perspectives was an appreciation of budgetary constraints, noted by both public and policy stakeholders. In the UK, LAs are funded by local taxes and central government grants, and have been subject to increasing financial pressures [72, 73]. Central government funding of LAs in England has fallen by 75% since 2010 [74], disproportionately affecting disadvantaged areas most dependent on central government grants [75]. LAs are therefore operating under ‘austerity localism’, where funding has been squeezed across a decade in which the political narrative has been about empowering local communities and their elected governments [76, 77]. These constraints both underline the need for research in the areas highlighted by the study - and make clear that more and better evidence is not sufficient to enable effective local action.

## Conclusions

Action at local level is integral to the delivery of health-centred climate policy. Our study sought to identify priority areas for research to support decision making by local government in England. Building on advice from policy stakeholders and members of the public, we identified a set of research priorities relating to public understanding, public acceptability of local actions and economic evaluation. We then assessed whether there was existing UK evidence that addressed these priorities. For each priority area, the study found a lack of evidence, and this was particularly marked for evidence on the economic implications of local climate action. There was also a dearth of evidence on the equity dimensions of local action. This includes evidence on whether and how public understandings and acceptability are related to and shaped by wider social inequalities and on potential inequalities in the economic costs and benefits of climate actions.

The study underlines the need for investment in research to support local action on health and climate change. Feedback from public and policy stakeholders also made clear that locally-tailored evidence is not the only, and is potentially not the major, barrier to local action. Stakeholders pointed to national/local governance structures and the wider impacts of a decade-long squeeze on LA budgets as major inhibitors of local action. Enhanced evidence portfolios to support action by LAs on health and climate change need to be part of a wider shift of resources to remedy a decade of austerity, and enable local government to deliver on its mandate to protect the public from the health impacts of climate change.

### Supplementary Information

**Additional file 1.** Policy Document Review – Methods.

**Additional file 2.** Directors of Public Health Survey.

**Additional file 3.** Policy Document Review – Results.

**Additional file 4.** Findings from Directors of Public Health Survey.

**Additional file 5.** Evidence Review and Inclusion/Exclusion Criteria.

## Data Availability

Full details of the study methods (including the online survey of DsPH), analyses and findings (including feedback from the public involvement panel) are included in this published article and its supplementary information files. The supplementary files can also be accessed at https://www.york.ac.uk/healthsciences/research/public-health/projects/research-priorities-on-climate-change/.

## Abbreviations

DsPH: Directors of Public Health
IMD: Index of Multiple Deprivation
ICB: Integrated Care Board
ICS: Integrated Care System
LA/LAs: Local authority/local authorities
UK: United Kingdom

## Footnotes

1 In England, London boroughs and other metropolitan areas (e.g. Birmingham, Manchester) operate as single authorities responsible for all council services in their area while smaller district councils share responsibilities with the larger county councils into which they are grouped (see Sandford, M. (2022). Local government in England: Structures. London: House of Commons Library (https://commonslibrary.parliament.uk/research-briefings/sn07104/)).

2 Public Health England was replaced by UK Health Security Agency UKHSA and Office for Health Improvement and Disparities (OHID) in 2021.

3 IMD is based on a range of indicators grouped into seven distinct domains: Income, Employment, Education, Skills and Training, Health and Disability, Crime, Barriers to Housing and Services, Living Environment.
