# Supplementary material for: Priorities for research to support local authority action on health and climate change: a study in England
Source: BMC Public Health. 2023 Oct 10;23:1965. doi: 10.1186/s12889-023-16717-1 (PMC10566048; doi:10.1186/s12889-023-16717-1)
Supplement: Supplementary file 1 — Additional file 1. Policy Document Review – Methods. [file 12889_2023_16717_MOESM1_ESM.docx]

*Supplementary File 1.*

**Policy Document Review – Methods**

## **Policy Document Selection**

The review sought to identify both local views of what is important with regards to health and climate change and the broader national perspective of what is considered important at the local level. Policy documents for this review were therefore split into two tiers.

*Tier one.* Climate action plans, and related documents, from local authorities made up one tier. These were supplemented by numerous city level climate strategies. Together, these plans demonstrate buy-in to action on health and climate change at the local level.

Local authority climate action plans were sourced from the CAPE online open-source database (CAPE, 2022); at the time of the review (add date), 83% of all local authorities had produced some form of plan or strategy. The city climate strategies were sourced individually following a thorough online search.

*Tier two.* The second tier was made up of national or broader documents including: the *National Assessment of Flood Risk* for England (Environment Agency, 2009) and the *Living Better with a Changing Climate* (Environment Agency, 2021) report to ministers, both from the Environment Agency; and the Climate Change Committee’s *Local Authorities and the Sixth Carbon Budget* report *(Climate Change Committee, 2020);* the UK Government’s *Levelling Up White Paper (HM Government, 2022)*;.

Due to time constraints, the choice of policy documentation was inevitably selective; a longer project would have enabled a more rigorous approach to selection.

## **Searching Policy Documents**

## *What counts as a “gap”?*

Given the difficulty of searching for “gaps” in a corpus, the research team collaboratively defined the object of the search, producing the following hierarchy:

1. Stated research priorities and evidence gaps (where the documentation itself provided accounts of what was missing in current evidence)
2. References to health (to explore the ways in which health is discussed within the policy documents)
3. The local authority sectors, if any, mentioned in relation to health, as these provide a broader context for health concerns and possible local action to deal with them.
4. Potential research priorities based on a framework derived from previous research in the area, namely Whitehead and Dahlgren (1991) and Haines and Ebi (2019).

## *Search terms*

All documents (stored as pdfs) were uploaded into programming software R (R Core Team, 2022), using functions from the base package and the *tabulizer* package (Leeper, 2022). The *tabulizer* package enabled a large variety of formatting within the pdfs to be uploaded into a clean and consistent dataframe. This dataframe could then be searched using the keyword-in-context (KWIC) functions within the *quanteda* R package (Benoit et al., 2018). KWIC functions enable a search of key words while also extracting a window of words around the keyword, allowing researchers to view the context of a particular term or search for co-occurring keywords. This opened up the possibility of searching for co-occurring health, climate change, sectoral, and other key terms.

Adapted from the hierarchy above, and developed iteratively when searching within the data and in line with other research on climate change and health (Dasandi, Graham, Lampard, & Mikhaylov, 2021; Romanello et al., 2022), search strings were built for each potentially relevant facet:

**Health:** "health", "illlness", "fatal", "mortal", "morbid", "hospital","doctor", "patient", "death", "disease", "fever","epidem","pandemic","medic","injur", "nutrition", "starv", "diagnos", "stress", "wellbeing", "well-being", "syndrom", "clinic", ” mental", "psycholog", "emotion", "covid", "coronavirus", "endemic”

**Climate change** (used within the nationally-oriented policy documents that weren’t solely concerned with climate change): “climate”, “warming”

**Climate change-related exposure:** "pollut", "flood", "heat", "erosion", "drought", "storm"

**Research priority/gap:** "investigat", "evidenc", "map", "examin", "consider", "probe", "question", "study", "inspect", "scrutinize"

**Sectoral:**

- *Business*: “commerc”, “business”, “industr”, “manufact”
- *Transport*: “transport”, “cycl”, “walk”, “road”, “travel”, “car”, “vehic”, “bus”, “rail”, “journ”
- *Housing*: “housing”, “homes”, “stock”, “affordab”, “development”, “energy_efficienc”, “regenerat”, “retrofit”, “insulat”, “building”
- *Agricultural*: “agricult”, “forest”, “livestock”, “farm”, “ammonia”, “soil”, “land_change”
- *Waste*: “recyc”, “landfill”, “waste”, “dispos”
- *Employment*: “employ”, “jobs”

## *KWICS and dataframes*

Multiple dataframes were then created searching for KWICs with a 30-word window around these keyword categories. These were explored to further refine the categories. Other sized windows were explored but it was found that the 30-word window captured enough words to pick up meaningful connections between words, along with the context carrying that meaning.

For each of the stages in the hierarchy outlined [above](#_What_counts_as) a dataframe was constructed, made up of KWICs. At stage one, this consisted of searching for research priority/gap keywords. At stage two, this consisted of searching for health keywords. At stage three, this consisted of searching for sectoral references and skimming through for relevant passages. Stage four, as a final iterative process, involved searching more generally through the documentation for passages that might be relevant across these keyword categories. This included aspects noted as important within previous literature on health and climate change.

Once constructed, these dataframes investigated in more detail. Due to the way in which the dataframes were constructed (i.e. using document IDs), it was possible to move easily back and forth between the dataframes and the original data, providing the opportunity to seek more context.

## **References**

Benoit, K., Watanabe, K., Wang, H., Nulty, P., Obeng, A., Müller, S., & Matsuo, A. (2018). quanteda: An R package for the quantitative analysis of textual data. *Journal of Open Source Software, 3*(30), 774.

CAPE. (2022). UK Council Climate Action Plans Database. Retrieved from <https://data.climateemergency.uk/about/>. Retrieved May 1 2022 <https://data.climateemergency.uk/about/>

Climate Change Committee. (2020). Local Authorities and the Sixth Carbon Budget. Retrieved from <https://www.theccc.org.uk/publication/local-authorities-and-the-sixth-carbon-budget/>

Dasandi, N., Graham, H., Lampard, P., & Mikhaylov, S. J. (2021). Engagement with health in national climate change commitments under the Paris Agreement: a global mixed-methods analysis of the nationally determined contributions. *The Lancet Planetary Health, 5*(2), e93-e101.

Environment Agency. (2009). Flooding in England: A National Assessment of Flood Risk. Retrieved from <https://www.gov.uk/government/publications/flooding-in-england-national-assessment-of-flood-risk>

Environment Agency. (2021). Living Better with a Changing Climate: Report to Ministers under the Climate Change Act Retrieved from <https://www.gov.uk/government/publications/climate-adaptation-reporting-third-round-environment-agency>

Haines, A., & Ebi, K. (2019). The imperative for climate action to protect health. *New England Journal of Medicine, 380*(3), 263-273.

HM Government. (2022). Levelling Up the United Kingdom. Retrieved from <https://www.gov.uk/government/publications/levelling-up-the-united-kingdom>

Leeper, T. J. (2022). tabulizer: Bindings for Tabula PDF Extractor Library (Version 0.2.3.). Retrieved from <https://github.com/ropensci/tabulizer>

R Core Team. (2022). R: A language and environment for statistical computing. Vienna, Austria: R Foundation for Statistical Computing. Retrieved from <https://www.R-project.org/>

Romanello, M., Di Napoli, C., Drummond, P., Green, C., Kennard, H., Lampard, P., . . . Costello, A. (2022). The 2022 report of the Lancet Countdown on health and climate change: health at the mercy of fossil fuels. *The Lancet, 400*(10363), 1619-1654. doi:<https://doi.org/10.1016/S0140-6736(22)01540-9>

Whitehead, M., & Dahlgren, G. (1991). Policies and strategies to promote social equity in health. *Stockholm: Institute for Future Studies*.
