# Supplementary material for: Priorities for research to support local authority action on health and climate change: a study in England
Source: BMC Public Health. 2023 Oct 10;23:1965. doi: 10.1186/s12889-023-16717-1 (PMC10566048; doi:10.1186/s12889-023-16717-1)
Supplement: Supplementary file 2 — Additional file 2. Directors of Public Health Survey. [file 12889_2023_16717_MOESM2_ESM.docx]

*Supplementary File 2.*

**Directors of Public Health Survey**

## **Project Overview**


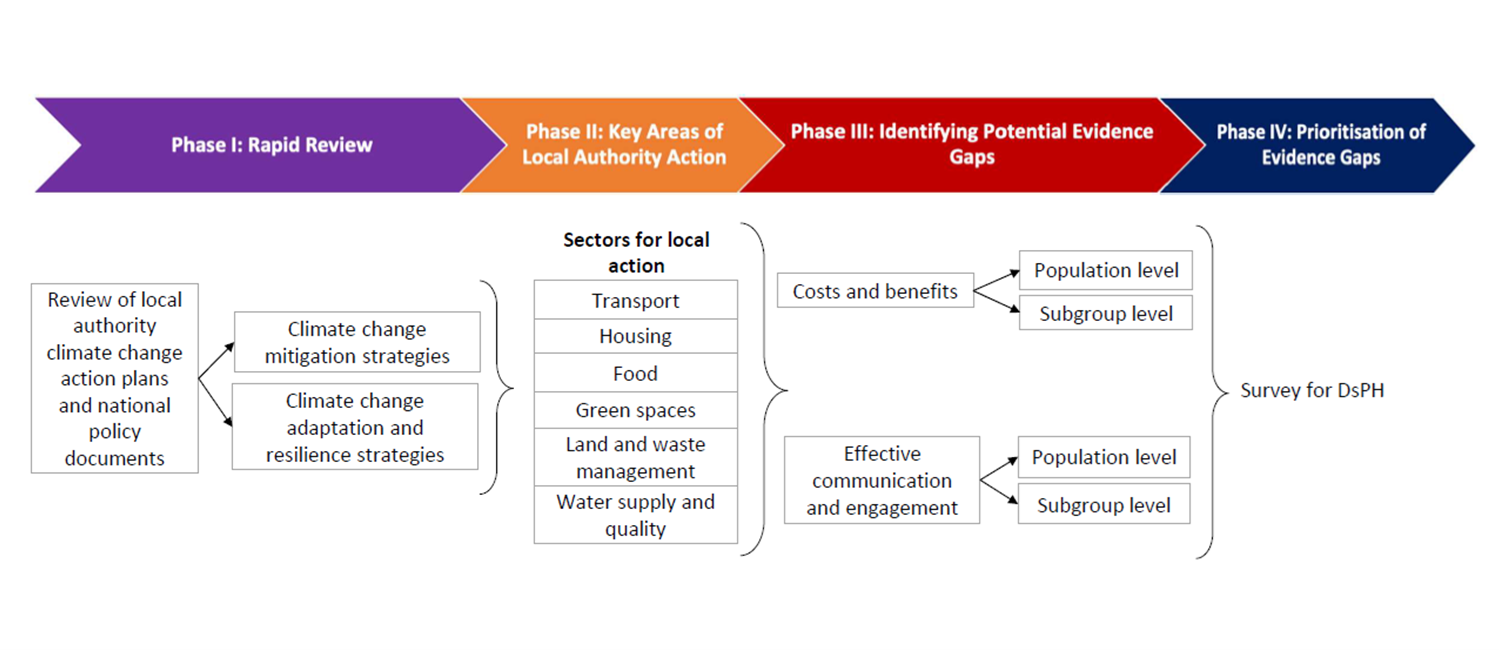


## **Invitation to Directors of Public Health**

Dear Director of Public Health,

**NIHR research to support action by local authorities on climate change and health**

We invite you to take part in a survey to identify evidence gaps that need to be filled if local authorities are to deliver an effective public health response to climate change.

The project is one of three projects funded by NIHR, the UK’s largest funder of public health research, to understand more about the evidence gaps that may be holding back local action on climate change and health. The project at the University of York is being led by Professor Hilary Graham, a public health researcher, and Professor Laura Bojke, a health economist, with support from Dr. Dagmar Zeuner, Director of Public Health at the London Borough of Merton. Other teams are based at the University of Lancaster and the London School of Hygiene and Tropical Medicine.

**Why are we focusing on action by local authorities?** Climate change is a global process – but its effects are local. Local authorities have a key role to play in both climate mitigation (reducing the greenhouse gas emissions driving climate change) and adaptation (adapting to changes that can no longer be prevented). Through the services they provide and the powers they have, local authorities play a key role in protecting people’s health from climate change, while maximising the health co-benefits of climate action. The diagram [here](#_Climate_change_impacts) illustrates how climate change can impact on people's health, and how local authority action – for example, through planning, housing, and transport policies – can influence these impacts.

**Why are we contacting Directors of Public Health?** The survey is being sent to DsPH because of their responsibilities for health improvement and health protection in their local authority. We have compiled a list of DsPH from Public Health England and are emailing everyone on this list. We are also including information on the survey in the ADPH newsletter.

**What does the survey cover (it takes about 5 mins to complete)?** The survey seeks your views on areas where you consider more evidence is needed to inform local-level action on climate change and health. The survey optionally asks for the name of the local authority you represent. This information is requested so that we can analyse differences in local authority priorities for research by various local-level factors, such as population density and IMD profile. Once the local-level information has been added to the dataset, the name of local authority will be removed. This means the data will be anonymous and no personal data, names or local authority contact details will be identifiable.

We very much hope you will take part in the survey by Friday, 26th August 2022. To access the survey, please click the arrow below.

Project updates will be shared with DsPH via the ADPH in due course.

Yours sincerely,

Hilary Graham and Laura Bojke

## **Survey: Local Authority Climate Change and Health**

**Introduction**

Thank you for your interest in completing the DsPH survey to support action by local authorities on climate change and health. It has ethical approval from the University of York Department of Health Sciences Research Governance Committee (ref HSRGC/2022/516/F).

Why are we conducting this survey? Local authorities play a key role in protecting people’s health from climate change. This includes climate mitigation (reducing the greenhouse gas emissions driving climate change) and adaptation (adapting to changes that can no longer be prevented).

Taking part in the survey is entirely voluntary. It takes about 5 mins to complete.

What does the survey cover? The survey seeks your views on areas where you consider more evidence is needed to inform local-level action on climate change and health. The survey also asks which local authority you represent. This information is collected so that we can analyse differences in local authority priorities for research by various local-level factors, such as population density and IMD profile. Once the local-level information has been added to dataset, the name of local authority will be removed. The data will be anonymous, and no personal data, names or local authority contact details will be identifiable.

To identify a preliminary list of evidence gaps and research priorities, we reviewed UK climate change-related policy documents (both local and national) and noted potential evidence gaps relating to action on climate change and health relevant to local authorities (click here for details).

These gaps and priorities fell into two broad areas: public communication and engagement, and economic implications. In the following questions, we ask for your feedback on these two areas.

How will the survey information be used? Findings will help to inform priorities for NIHR research on climate change and health. Project updates will be shared with DsPH via the ADPH in due course. A summary of findings will form part of the project report published on the NIHR website and associated outputs (e.g. in a research journal).

More information on how the survey data is stored and used is available here.

If you have any questions about the survey, please contact Hilary Graham at the University of York (hilary.graham@york.ac.uk).

*End of Block: Introduction and consent*

*Start of Block: Local authority*

**Please indicate if you do or do not represent a local authority**

> I **do** represent a local authority

> I **do not** represent a local authority

**Please indicate which local authority you represent.** *This information is optionally requested so that we can analyse differences in local authority priorities for research by various local-level factors, such as population density and IMD profile. Once the local-level information has been added to the dataset, the name of local authority will be removed. This means the data will be anonymous and no personal data, names or local authority contact details will be identifiable.*

____________________________________________________

*End of Block: Local authority*

*Start of Block: Communication and Engagement*

**Communication and Engagement**

Community engagement is central to effective local action on the health impacts of climate change.

Please select up to three areas from the following list where, in your view, more evidence is needed by your local authority on ways to engage the public in local action to mitigate and adapt to the health impacts of climate change.

More evidence is needed on:

▢ Public understandings of climate change and its impacts on people’s health

▢ The effectiveness of different ways of consulting with local communities

▢ The public acceptability of local actions (e.g. low traffic neighbourhoods)

▢ Best practice in engaging with local businesses

▢ Other, please specify: __________________________________________________

*End of Block: Communication and Engagement*

*Start of Block: Populations: Communication and Engagement*

Please select up to three groups or communities where, in your view, more evidence is needed by your local authority on effective ways to engage the public in local level actions to mitigate and adapt to the health impacts of climate change.

▢ All communities

▢ Communities from different ethnic and cultural backgrounds

▢ Different age groups (e.g. children; older people)

▢ Different gender and sexual identities (e.g. women, men, LGBT+)

▢ Different income groups (e.g. richer and poorer households)

▢ Communities facing barriers to decent housing and local services

▢ People with long-term physical and mental health conditions

▢ Other, please specify: __________________________________________________

*End of Block: Populations: Communication and Engagement*

*Start of Block: Economic implications*

**Economic implications**

There are economic (cost) implications of actions to mitigate and adapt to the health impacts of climate change. It may be important to understand how costs and benefits are generated, and for which sectors, as well as to quantify the costs and benefits for different groups and communities.

Please select up to three areas, where, in your view, more evidence is needed by your local authority to understand the economic (cost) implications of actions to mitigate and adapt to the health impacts of climate change.

▢ Evidence on the health-related costs and benefits of investing in climate change mitigation and adaptation activities

▢ Evidence on the health and non-health-related costs and benefits of investing in climate change mitigation and adaptation activities

▢ Information on the short, medium, and long-term budgetary implications of climate change mitigation and adaptation activities

▢ Best practice evidence on policies to financially incentivise local businesses to adopt climate change mitigation and adaptation activities

▢ Best practice evidence on policies to financially incentivise individual residents to adopt climate change mitigation and adaptation activities

▢ Other, please specify: __________________________________________________

*End of Block: Economic implications*

*Start of Block: Sectors: Economic implications*

Please select up to three sectors where, in your view, more evidence is needed by your local authority to understand the economic (cost) implications of actions to mitigate or adapt to the health impacts of climate change.

▢ Active travel infrastructure and active lifestyles

▢ Green spaces, green networks, green infrastructure

▢ Healthier diets and sustainability of food supply

▢ Built environment, building design, healthy homes schemes

▢ Land and waste management

▢ Air quality, air pollution, clean air zones

▢ Disaster management

▢ Water quality and supply

▢ Crossover between multiple sectors, please specify: _________________________

▢ Other, please specify: _______________________________

*End of Block: Sectors: Economic implications*

*Start of Block: Populations: Economic implications*

Please select up to three groups or communities where, in your view, more evidence is needed by your local authority on the differential economic impacts of local level actions to mitigate or adapt to the health impacts of climate change.

▢ All communities

▢ Communities from different ethnic and cultural backgrounds

▢ Different age groups (e.g. children; older people)

▢ Different gender and sexual identities (e.g. women, men, LGBT+)

▢ Different income groups (e.g. richer and poorer households)

▢ Communities facing barriers to decent housing and local services

▢ People with long-term physical and mental health conditions

▢ Other, please describe: _________________________

*End of Block: Populations: Economic implications*

*Start of Block: Qualitative Questions*

In your opinion, are there other key evidence gaps and research priorities which, if addressed, will help your local authority protect people's health from climate change?

________________________________________________________________

________________________________________________________________

________________________________________________________________

________________________________________________________________

________________________________________________________________

*End of Block: Qualitative Questions*

## **Climate change impacts on human health**


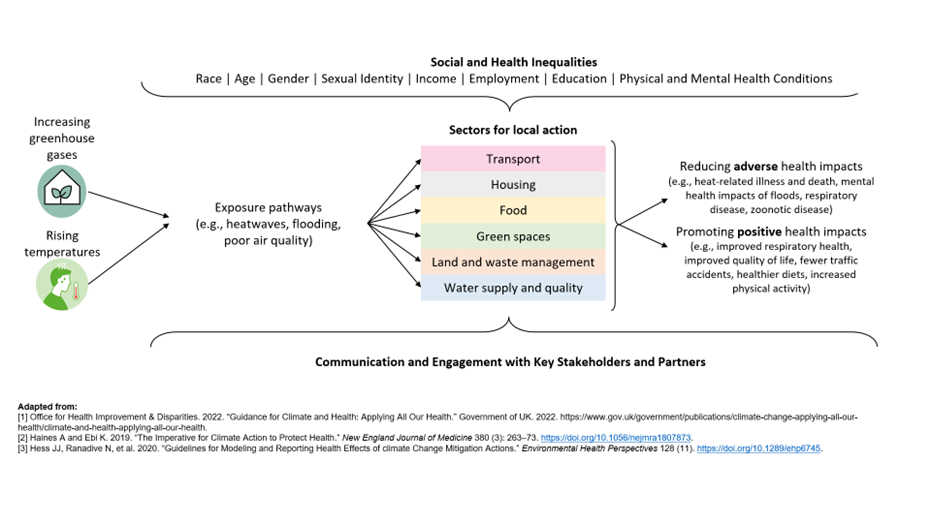


## **Privacy Notice**

**Privacy notice – NIHR project on local authority-level research priorities on climate change and health**

Hilary Graham, Laura Bojke, Joy Adamson

This privacy notice sets out the ways in which data gathered through the survey of Directors of Public Health will be stored and used by the University of York. The survey is part of research to support action by local authorities on climate change and health. The privacy notice also sets out how long we keep the survey data and what rights you have in relation to your data under the General Data Protection Regulation (GDPR) and the Data Protection Act 2018.

**1) Who is responsible for the data?**

For the purposes of this privacy notice, University of York is a Data Controller as defined in the GDPR. We are registered with the Information Commissioner’s Office and our entry can be found here. Our registration number is: Z4855807.

We have an appointed Data Protection Officer who can be contacted on the following address: Data Protection Officer, University of York, Heslington, York, YO10 5DD, UK; email: dataprotection@york.ac.uk.

We will place any updates to this privacy notice on this web page.

**2) Who is conducting the research?**

The research is conducted by researchers at the University of York. It forms part of a project funded by NIHR that is mapping local authority-level research priorities on climate change and health.

**3) What are the objectives of the research and the purpose of using the data?**

The University of York’s charter is for the advancement of learning and knowledge through teaching and research. We will use the data collected to gain an overview of the views of Directors of Public Health (DsPH) on evidence gaps that need to be filled if local authorities are to deliver an effective public health response to climate change. We will also explore whether their views differ depending on various local factors, such as deprivation in their local community and population density. This will help us advise NIHR on the evidence gaps that may be holding back local action on climate change and health.

**4) Where do we get the data from?**

For this research, the data will come from the survey of Directors of Public Health.

**5) What data do we hold?**

The data we hold are the answers to questions we ask in our survey of DsPH. No personal data (e.g. name and title) are collected or held.

For those who participate, the survey optionally asks which local authority you represent. This information is requested so that we can analyse differences in local authority priorities for research by various local-level factors, such as population density and IMD profile. Once the local-level information has been added to dataset, the name of local authority will be removed.

This means the data will be anonymous; no personal data, names or local authority contact details will be identifiable. We will also not be able to identify which recipients of our invitation to participate in our survey did not do so.

**6) What is our legal basis for processing the data?**

The GDPR requires us to establish a legal basis for processing data from the survey of DsPH. For the purpose of this privacy notice, the processing is necessary for the performance of a task carried out in the public interest. Our public task functions are as set out in our University charter.

**7) Who do we share the data with?**

Permitted employees of the University of York working on the research project will use the data. Although we do not anticipate the requirement to share the data with third parties, should this be required disclosures would be made only in full accordance with data protection legislation and only where necessary. You will be advised of such disclosures through this privacy notice unless exceptional circumstances apply.

We will disseminate the results of the survey via reports, presentations and academic papers. In these publications, all results will be reported in aggregate format in a way that prevents answers from being traced back to individual local authorities.

**8) How do we keep the data secure?**

The University of York takes information security extremely seriously and has implemented appropriate technical and organisational measures to protect personal data and special category data. The data are stored on the University of York’s secure data system.

Access to information is restricted on a need-to-know basis and security arrangements are regularly reviewed to ensure their continued suitability. For further information see, https://www.york.ac.uk/it-services/security/.

**9) Will we transfer the data internationally?**

No, we will not transfer any data out of the UK.

**10) How long will we keep the data for?**

Data will be retained for the duration of the funding of the project and until outputs from the research have been published. Data will be destroyed safely, in accordance with the Data Protection Laws.

**11) What rights do you have in relation to your data?**

Under the General Data Protection Regulation, you have a right of access to your data. We will retain the local authority identifier, where it has been shared, for 5 working days after you have submitted the survey to enable you to exercise a right to rectification and erasure of the data you submitted.

**12) Questions or concerns**

If you have any questions about this privacy notice or concerns about how the data is being processed, please contact the University’s Data Protection Officer at dataprotection@york.ac.uk.

**13) Right to complain**

If you are unhappy with the way in which the University has handled the data, you have a right to complain to the Information Commissioner’s Office. For information on reporting a concern to the Information Commissioner’s Office, see www.ico.org.uk/concerns
