# Supplementary material for: Priorities for research to support local authority action on health and climate change: a study in England
Source: BMC Public Health. 2023 Oct 10;23:1965. doi: 10.1186/s12889-023-16717-1 (PMC10566048; doi:10.1186/s12889-023-16717-1)
Supplement: Supplementary file 3 — Additional file 3. Policy Document Review – Results. [file 12889_2023_16717_MOESM3_ESM.docx]

*Supplementary File 3.*

**Policy Document Review – Results**

### **Overview**

The overriding finding from the policy document review was the small amount of content relating to health. This was the same across local climate action plans and broader national documents.

In relation to the hierarchy presented regarding what counts as “gaps and priorities” in Supplementary File 1, this meant that most of the references from the documents came via 2) references found that somehow linked to health, 3) sectors mentioned in relation to health mentions, and 4) potential research priorities from previous research in the area of climate change and health that maps on to the discourse of the policy documentation. There were very few stated research priorities and evidence gaps in the area of climate change and health more specifically (1).

Areas that did emerge included those relating to financial strategy and decision making, vulnerabilities and equity in action, communication and engagement, and interconnections between sectors. These will be outlined in more detail below, with examples taken from documentation.

### **Financial strategy and decision making**

Various themes arose in relation to long- and short-term cost-benefit analyses. Items in this area included the economic impacts of climate hazards at local levels, for example:

*There is a gap in understanding of the quantified economic impacts of climate hazards at a local level. We recommend quantification of economic impacts of climate hazards in Bristol is undertaken to help build a business case for action. This includes the direct impacts, impacts on revenues and sales, impact on productivity, and impacts on resources, production and supply chains. This would aid understanding of the economic viability of climate adaptation and assist in stimulating funding for climate adaptation measures (Bristol City Council, 2020)*

Another item related to whole-life costing:

*Sustainable procurement is a way of buying goods, services, work and utilities that meets our value for money expectations whilst generating benefits to our communities and minimising damage to our environment. Traditional procurement has focused upon value for money considerations. The aim of sustainable procurement is to take greater account of social and environmental considerations when purchasing or deciding to purchase with the goal of reducing adverse impacts upon the health of individuals and communities, social impact and environmental impact thereby bringing greater benefits to the community at large. Procuring in a sustainable manner can achieve greater value for money as ‘****whole life costs****’ will be taken into account. Taking such a procurement approach can also stimulate the market for sustainable technologies, improve our communities’ standard of living, improve health and the environment and save money. [...] The Procurement team at Bolsover DC & NEDDC have been providing a* ***cradle to grave*** *Procurement process via the In-tend system since the team was established in May 2018 (Bolsover District Council, 2021)*

*Reducing Financial Costs. Although adaptation and mitigation action may be expensive initially, if whole life costs are considered, often such measures tend to be cheaper than business as usual in the long term. Short term costs are often worth the savings across multiple departments and levels in the long term. These indirect or long-term savings are important to local authorities striving to provide value for money to the taxpayer (Sefton Metropolitan Borough Council, 2019)*

*Employing these measures will comes at an increased cost therefore it is essential that consideration is given to a Whole Life assessment to support any capital investment; which assesses the total cost of an asset over its whole life. It takes account of the initial capital cost, as well as operational, maintenance, repair, upgrade and eventual disposal costs. Whole life costing can also factor in related income streams, where appropriate The Scottish Future Trust have designed a Whole Life Appraisal tool to assist the public sector make informed decisions to optimise a built asset’s whole life performance (East Ayrshire Council, 2022)*

Another investment-related item came in the form of ideas around “invest-to-save”:

*Funding the response Tackling the climate emergency will require significant investment at all levels of society. Dorset Council alone will need to invest many mil- lions of pounds (over £100m) over the next 20-30 years just to be- come a Carbon Neutral Council. Many measures will have a financial return on investment, but many may not. However, many will have wider health and economic benefits which can be realised. As further work is done to draw up detailed implementation plans for our buildings, vehicles, and energy infrastructure, we will need to develop a detailed financial strategy. It is a challenging, financial time for us all, especially Local Authorities. We will develop a finance strategy to enable us to deliver this climate emergency response by identifying and implementing ways for our finance department to actively support climate change action. This includes establishing invest-to-save schemes, such as our transformation fund, capital receipts through asset rationalisation, and building into our capital programme (Dorset Council, 2022)*

Economic and co-benefits cases were made for diversifying practice:

*Establish incentives (policy and funding) to support investment by public and private organisations to deliver exemplar low carbon, climate resilient improvements to their estates (Manchester City Council)*

*Build capacity to partner in research, demonstration, pilot and pathfinder projects. Energy requirements from buildings, transport and industry coupled with distributed energy generation will change the way the electricity grid operates. Flexibility, batteries and smart appliances combined with price or carbon signals will shift consumption from peak demand times towards periods when renewable energy is available. New business and finance models need to be developed in ways that are fair and affordable, local authorities are useful partners for pilot projects because they have access to public estate and housing where schemes can be trialled and monitored (Climate Change Committee, 2020)*

Economic incentives were also foregrounded:

*Establish incentives (policy and funding) to support investment by public and private organisations to deliver exemplar low carbon, climate resilient improvements to their estates (Manchester City Council, 2016)*

### **Vulnerabilities and equity in action**

Numerous forms of vulnerability and inequality emerged in the policy documents, some of which mentioned health, others with clear health implications. One example of this relates to the impacts of climate change on healthcare within care homes:

*Research: Given the existing vulnerability of elderly people to the impacts of climate change, undertake a study of climate resilience measures in care homes and other health care facilities (South Gloucestershire Council, 2020)*

Others focused on inequalities at a point further upstream, looking for earlier local solutions:

*Work in partnership with our communities to identify local travel and transport solutions and to ensure a fair and just transition to a carbon neutral East Lothian; explore options for shared community transport options, particularly for our rural communities. Community involvement in decision-making over local transport and travel solutions (East Lothian Council, 2019)*

*Climate change will exacerbate existing environmental inequalities, since some groups will be more affected by climate risks or have less capacity to prepare for them. We want to ensure no group is left behind by climate change in line with the government’s levelling up commitments. We will do this by: • Trialling different approaches to flood warning and informing, through stronger community engagement, use of third parties and new technologies • Understanding how our incident management service will need to evolve to cope with the changing scale, geography and nature of climate related incidents, including surface water flood risk, and the dynamic needs of a changing and increasingly diverse population • Working with our public health partners to better understand and integrate thinking on how we can reduce inequalities as a result of climate change […] Expand work with partners for a holistic approach to reducing poor health and wellbeing and reducing inequalities resulting from climate change. Our activities contribute to protecting health and wellbeing and reducing inequalities in a changing climate (Environment Agency, 2021)*

Discussions of fuel poverty also highlighted important inequalities:

*Fuel poverty – It is clear that the effects of Covid-19 will lead to an economic recession, and this holds the potential to worsen existing poverty and deprivation within our communities. We have the power to tackle this by leveraging our influence to lobby for funding to improve energy efficiency in the domestic housing sector and by using our existing partnerships to carry out the work required, to a high standard and at a competitive rate. Such efforts can provide multiple benefits, by decarbonising hard-to- treat housing stock, increasing disposable income (by reducing fuel bills), avoiding health problems from poorly-heated homes, and improving people’s mental wellbeing (by alleviating stress and financial hardship) (Wakefield Council, 2019)*

*By improving energy efficiency, we can also ensure that everyone, even the most vulnerable, can afford to stay warm enough in their home. In 2018, 4,114 households in St Albans (6.9%) experienced fuel poverty. This means that these residents may not have been able to keep their homes warm enough at a reasonable cost. This happens when homes are inefficient at retaining heat, income is low, and fuel costs are high. As a result, people can find themselves having to make choices between warmth or food. Over time this can affect physical and mental health. In 2018/19 the East of England had a higher excess winter death index (15.2%) than the England average (14.2%) for the same period (St Albans City and District Council, 2019)*

### **Communication and engagement**

One area that emerged, with overlap in previous literature, is communication with the general public and communities about climate change and its impact on health. A central aspect of this was how to build stronger understandings ?within local communities and a higher level of engagement:

*There is currently a lack of understanding at a community level of how climate hazards may impact people and communities. A wide ranging community-level programme of engagement, building on existing initiatives would support building resilience for communities. This would support community preparedness for action when a climate hazard event occurs (Bristol City Council, 2020)*

Different plans and documents had different orientations of what communication might entail:

*Keep communicating: Provide advice and information for residents and businesses on energy efficiency and low-carbon heating options. Raising awareness of the transition needs to be undertaken in sequence with practical support and options for action, so that people are supported. Working with delivery partners and community-based organisations to promote what works locally through Green Buildings Week (Climate Change Committee, 2020)*

*Develop full communications and marketing plan (increase in awareness of issues and participation in environmentally-positive behaviours)… Introduce programme of behaviour change initiatives to encourage adoption of environmentally-positive lifestyle changes (Blackpool Borough Council, 2019)*

There was also a focus on methods by which people can be engaged:

*Communication will certainly be key to ensuring that Dundee is resilient to climate change. Whether it be engaging with communities to co-design resilient neighbourhoods or helping schools and businesses and communities prepare for future risks to health or climate hazards and possible interruptions to essential services, clever and effective methods of communication will be essential.* ***ACTION R.12:*** *Develop adaptation engagement tools to support community capacity building, including visual and interactive tools, workshops and collaboration with community organisations (Dundee City Council, 2019)*

Some were interested in barriers to effective communication and engagement:

*Researching and evidencing the specific local physical, mental, and perceived barriers to active travel in different circumstances and in different parts of the district in order to more effectively target and support engagement, education, incentives and interventions (Somerset West and Taunton Council, 2020)*

Others foregrounded the need to communicate with different groups:

*Significant public engagement will take place across the city to ensure that residents have their say on actions to tackle climate change and improve their neighbourhoods, including topics such as food, energy, transport, natural environment, health and climate justice. This will utilise innovative engagement methods, while working collaboratively with the arts community, academia, and civil society. To support this, the City will roll out climate conversations [action 2] throughout Glasgow and enable residents to access information and make recommendations for change. In order to connect with key stakeholders, we will engage with our partners within the Community Planning Partnership and Glasgow Life. Engagement will continue to be undertaken with young people to include, amongst other topics, discussions about how proposed land use and connectivity policies can help to achieve a resilient and net zero carbon city by 2030, as part of the City Development Plan 2 and the Glasgow’s Transport Strategy. Communications and engagement about the climate and ecological emergency must also include work with Education Services and the curriculum for excellence, exploring opportunities for outdoor learning to mainstream climate change awareness and to help foster action (Glasgow City Council, 2020)*

### **Interconnections between sectors**

Another area emerging from the policy document review was the need for joined up action:

*Continue to develop the local evidence base and understanding of the benefits of joined-up action on health and climate change (Manchester City Council, 2016)*

*Work will be undertaken to better understand the likely impacts, on service providers and users and on the wider community, arising from changes in weather patterns, and action will be taken to reduce risk and increase resilience to the anticipated future effects of climate change. Prioritisation of action will be based on contexts where service users are most vulnerable and contexts where there is a direct link to existing organisational priorities, including biodiversity action planning, health promotion and flood risk management (East Dunbartonshire Council, 2016)*

This also emerged in the form of cascading impacts:

*Interdependencies between assets exist, but an overarching understanding of cascading impacts is lacking. It is recommended that work is undertaken to develop greater understanding of interdependencies between physical and non-physical systems. This should build on research done at a national level, for example by the National Infrastructure Commission and as part of the Climate Change Risk Assessment 3. We recommend a study to understand the potential climate risks through supply chains for organisations in the city (Bristol City Council, 2020)*

### **References**

Blackpool Borough Council. (2019). Blackpool’s Climate Emergency Action Plan. Retrieved from <https://data.climateemergency.uk/media/data/plans/blackpool-borough-council-559805e.pdf>

Bolsover District Council. (2021). Climate Change Update & Renewable Energy Tariff. Retrieved from <https://data.climateemergency.uk/media/data/plans/bolsover-district-council-38140ff.pdf>

Bristol City Council. (2020). Bristol One City Climate Strategy: Preliminary Climate Resilience Assessment. Retrieved from <https://www.bristolonecity.com/wp-content/uploads/2020/02/Preliminary-climate-resilience-assessment.pdf>

Climate Change Committee. (2020). Local Authorities and the Sixth Carbon Budget. Retrieved from <https://www.theccc.org.uk/publication/local-authorities-and-the-sixth-carbon-budget/>

Dorset Council. (2022). Climate and Ecological Emergency Strategy. Retrieved from <https://data.climateemergency.uk/media/data/plans/dorset-council-119e117.pdf>

Dundee City Council. (2019). Dundee Climate Action Plan. Retrieved from <https://data.climateemergency.uk/media/data/plans/dundee-city-council-5aef502.pdf>

East Ayrshire Council. (2022). East Ayrshire Climate Change Strategy - Outcome of Engagement Activity and Future Action. Retrieved from <https://data.climateemergency.uk/councils/east-ayrshire-council/>

East Dunbartonshire Council. (2016). Sustainability and Climate Change Framework 2016 – 2021. Retrieved from <https://data.climateemergency.uk/media/data/plans/east-dunbartonshire-council-c002680.pdf>

East Lothian Council. (2019). Climate Change Strategy 2020-2025. Retrieved from <https://data.climateemergency.uk/media/data/plans/east-lothian-council-f30fcfb.pdf>

Environment Agency. (2021). Living Better with a Changing Climate: Report to Ministers under the Climate Change Act Retrieved from <https://www.gov.uk/government/publications/climate-adaptation-reporting-third-round-environment-agency>

Glasgow City Council. (2020). Glasgow’s Climate Plan: Our Response to the Climate and Ecological Emergency. Retrieved from <https://data.climateemergency.uk/media/data/plans/glasgow-city-council-9be8bc9.pdf>

Manchester City Council. (2016). Manchester Climate Change Strategy 2017-50: Implementation Plan 2017-22. Retrieved from <https://data.climateemergency.uk/media/data/plans/manchester-city-council-7a09b33.pdf>

Sefton Metropolitan Borough Council. (2019). Sefton’s Climate Change Emergency Implementation Plan. Retrieved from <https://data.climateemergency.uk/councils/sefton-metropolitan-borough-council/>

Somerset West and Taunton Council. (2020). Carbon Neutrality and Climate Resilience Plan: Framework Document. Retrieved from <https://data.climateemergency.uk/media/data/plans/somerset-west-and-taunton-council-4a38145.pdf>

South Gloucestershire Council. (2020). Climate Emergency Strategy. Retrieved from <https://data.climateemergency.uk/media/data/plans/south-gloucestershire-council-de04afe.pdf>

St Albans City and District Council. (2019). Sustainability and Climate Crisis Strategy. Retrieved from <https://data.climateemergency.uk/media/data/plans/st-albans-city-and-district-council-aefff99.pdf>

Wakefield Council. (2019). Climate Change Action Plan. Retrieved from <https://data.climateemergency.uk/media/data/plans/wakefield-metropolitan-district-council-5c4d49d.pdf>
