# Supplementary material for: Priorities for research to support local authority action on health and climate change: a study in England
Source: BMC Public Health. 2023 Oct 10;23:1965. doi: 10.1186/s12889-023-16717-1 (PMC10566048; doi:10.1186/s12889-023-16717-1)
Supplement: Supplementary file 4 — Additional file 4. Findings from Directors of Public Health Survey. [file 12889_2023_16717_MOESM4_ESM.docx]

*Supplementary File 4.*

**Findings from Directors of Public Health Survey**

## Methods

In the survey, DsPH were asked to report their local authority (LA), allowing us to explore whether LA demographics affected DPH’s perceived research priorities. Specifically, we analysed the impact of population density, to explore whether priorities different in rural and urban areas, and index of multiple deprivation (IMD). IMD measures relative deprivation in small areas in England called lower-layer super output areas. The Indices are based on a range of indicators, grouped into seven distinct domains: Income, Employment, Education, Skills and Training, Health and Disability, Crime, Barriers to Housing and Services, Living Environment. Each Index represents a different aspect of deprivation.

Population density and IMD scores for each LA were derived from ONS data on the population profiles for LAs in England (1). The ONS data records the following information for each LA: population age, population density (people per square kilometre), proportion of school children, age groups, ethnicity, employment, industry, proportion of population in key worker occupations, and deprivation (IMD2019 scores).

In the ONS dataset, data is reported at level of district councils, boroughs or unitary authorities. For Public Health bodies, some LAs have entered into shared agreements, so one DPH resides over several LAs. When this is the case, IMD scores were derived as the mean of individual LAs weighted by the population size in each LA, and population density was derived by dividing the total population by the total surface area, for all relevant LAs. When DsPH reported working at county-level, the list of their LAs was obtained from the county website.

The LA demographics were used to conduct subgroup analyses of the survey results. For the sample of DsPH who reported their LA in the survey, we compared the answers submitted by DsPH working in LAs where IMD is less than that the national (England) average to those where it is greater. Furthermore, we compared the results for the 10 most deprived (lowest IMD) and least deprived (highest IMD) LAs, to explore whether research priorities differ in the LAs with more extreme demographics. This subgroup analysis was repeated for population density.

## Results

## *Sample description*

In the survey, 57 DsPH took part (38% response rate) and one non-DsPH, where 51 DsPHs reported which LA in England they represent. Of these 51 DsPH, 33 reported working in a single LA (district council, borough or unitary authority), 7 DsPH reported working in two or more LAs, while 11 reported working at county-level.

Of the 51 DsPH, 76.5% (39 DsPH) worked in LAs with a climate action plan; this is comparable to the overall proportion of LAs (74.4%) that have a climate action plan, suggesting the DsPH more likely to be engaged in climate action were not overrepresented in our sample.

**Table 1** compares demographics of the sample of LAs whose DsPH responded to the survey and England. The table indicates that the LAs of the surveyed DsPH were representative of England in terms of IMD (23.0 for the survey sample compared to 21.7 for England) but more populated than England on average, as the average number of people per square km was 3,082 compared to 432 in England. Eleven of the 51 LAs (21.6%) had population density lower than the national average.

Table 1. Demographics of the sample of LAs in the survey and the population (England).

|  | **Survey – full sample** | **Survey - disclosed LAs** | **England** |
| --- | --- | --- | --- |
| Number of LAs  (% of all LAs in England) | 58 (43%) | 51 (38%) | 135 (100%) |
| Total population  (% of total population in England) | Unknown | 22,724,000 (43%) | 53,012,456 (100%) |
| Average IMD score | Unknown | Mean: 23.0  Range: 10.2 to 42.4 | 21.7 |
| Proportion of LAs with lower IMD scores than the national average (%) | Unknown | 25 (49.0%) | 194 (61.8%) |
| Population density  (people per sq. km) | Unknown | Mean: 3,082  Range: 64 to 16,427 | 432 |
| Number of LAs with lower population density than the national average (%) | Unknown | 11 (21.6%) | 121 (38.5%) |

Overall, IMD and population density were not correlated in the sample who reported their LA (Pearson correlation coefficient 0.21). The distribution of the two characteristics is shown in Figure 1. The figure suggests a weak positive correlation between IMD scores and population density, with few LAs that have low population density and high IMD score (deprived rural areas).

Figure 1. IMD and population density for responders who reported their LA in the survey.


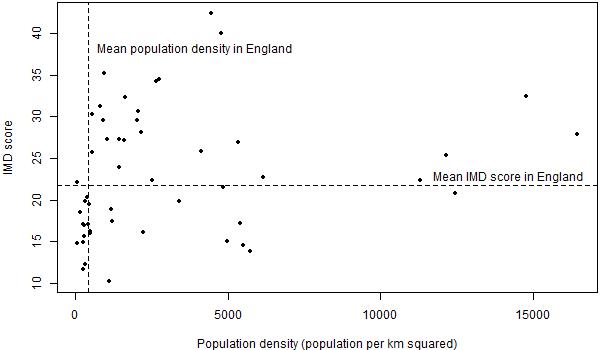


## *Summary of results*

The research priorities of DsPHs are summarised in Table 2. The top four research topics taken forward for rapid reviews were:

- Community engagement: the public acceptability of local actions (e.g. low traffic neighbourhoods);
- Community engagement: public understandings of climate change and its impacts on people’s health;
- Economic implications: the evidence on the health and non-health-related costs and benefits of investing in climate change mitigation and adaptation activities;
- Economic implications: short, medium and long term budgetary implications of climate change mitigation and adaptation activities.

Table 2. Top three research priorities

| **Survey question** | **Top three research priorities** |
| --- | --- |
| 1. Areas where more evidence is needed by your local authority on ways to engage the public in local action to mitigate and adapt to the health impacts of climate change | 1. The public acceptability of local actions (e.g. low traffic neighbourhoods) (84.5%) 2. Public understandings of climate change and its impacts on people’s health (74.1%) 3. Best practice in engaging with local businesses (48%) |
| 1. Groups or communities where more evidence is needed on effective ways to engage the public in local level actions to mitigate and adapt to the health impacts of climate change on health | 1. Different income groups (e.g. richer and poorer households) (65.5%) 2. Communities facing barriers to decent housing and local services (44.8%) 3. Communities from different ethnic and cultural backgrounds (41%) |
| 1. *Area* where more evidence is needed to understand the economic (cost) implications of actions to mitigate and adapt to the health impacts of climate change | 1. Evidence on the health and non-health-related costs and benefits of investing in climate change mitigation and adaptation activities (75.9%) 2. Evidence on the health and non-health-related costs and benefits of investing in climate change mitigation and adaptation activities (69%) 3. Best practice evidence on policies to financially incentivise local businesses to adopt climate change mitigation and adaptation activities (48%) |
| 1. Specific sectors where more evidence is needed to understand the economic (cost) implications of actions to mitigate and adapt to the health impacts of climate change | 1. Built environment, building design, healthy homes schemes (60.3%) 2. Healthier diets and sustainability of food supply (48.3%) 3. Active travel infrastructure and active lifestyles (38%) |
| 1. Groups or communities where more evidence is needed to understand the economic (cost) implications of actions to mitigate and adapt to the health impacts of climate change | 1. Different income groups (e.g. richer and poorer households) (65.5%) 2. Different income groups (e.g. richer and poorer households) (50%) 3. All communities (45%) |

## *Subgroup analysis*

Table 2 and Table 3 show the differences in the top two research priorities for each research question, in the 10 most and least deprived LAs, and the ten least and most densely populated LAs, respectively. The tables suggest three key differences:

1. Key research priorities in the most and the most deprived LAs differed for one survey question, about the sectors where more evidence is needed to understand the economic (cost) implications of actions to mitigate or adapt to the health impacts of climate change. The two sectors considered key research priorities overall were “healthier diets and sustainability of food supply” and “built environment, building design, healthy homes schemes”. However, in the 10 LAs with the lowest IMD (least deprived), the two key sectors were “active travel infrastructure and active lifestyles” and “green spaces, green networks, green infrastructure”. When comparing the local authorities with IMD below and above the national average, the top two priorities in both subgroups we identical to the sample overall, indicating that only the least deprived LAs differed in their views.
2. The least and most densely populated LAs indicated different research priorities relating to groups and communities where more evidence is needed on effective ways to engage the public in local level actions to mitigate and adapt to the health impacts of climate change on health. The groups and communities considered key research priorities overall were “different income groups (e.g. richer and poorer households)” and “communities facing barriers to decent housing and local services”. However, in the 10 LAs with the highest population density in the sample, the key groups and communities were “communities from different ethnic and cultural backgrounds” and “different age groups (e.g. children; older people)”. The 10 LAs with the lowest population density in the sample, also indicated that “communities from different ethnic and cultural backgrounds” were a key research priority. These differences were not sustained when comparing local authorities with population density below and above the national average, indicating that only the most densely populated LAs differed in their views.
3. The least and most densely populated LAs indicated different research priorities relating to groups and communities where more evidence is needed on differential economic impacts of these local level actions to mitigate or adapt to the health impacts of climate change on health. The groups and communities considered key research priorities overall were “different income groups (e.g. richer and poorer households)” and “communities facing barriers to decent housing and local services”. In the 10 most densely populated LAs, the priorities were “all communities” and “communities from different ethnic and cultural backgrounds”. When comparing the local authorities with population density below and above the England average, the top two priorities were the same as those for the sample overall, indicating that only the most densely populated LAs differed in their views.
4. Finally, when comparing key research priorities for the LAs with IMD or population density greater than the national average, the survey results did not change significantly.

Table 3. Differences in top two research priorities, by IMD score

| **Survey question** | **Top two research priorities** | | |
| --- | --- | --- | --- |
|  | **All LAs** | **LAs with 10 lowest IMD scores** | **LAs with 10 highest IMD scores** |
| 1. Areas where more evidence is needed by your local authority on ways to engage the public in local action to mitigate and adapt to the health impacts of climate change | The public acceptability of local actions (e.g. low traffic neighbourhoods) (84.5%) | Unchanged (80%) | Unchanged (100%) |
|  | Public understandings of climate change and its impacts on people’s health (74.1%) | Unchanged (60%) | Unchanged (70%) |
| 1. Groups or communities where more evidence is needed on effective ways to engage the public in local level actions to mitigate and adapt to the health impacts of climate change on health | Different income groups (e.g. richer and poorer households) (65.5%) | Unchanged (70%) | Unchanged (80%) |
|  | Communities facing barriers to decent housing and local services (44.8%) | Unchanged (40%) | Unchanged (70%) |
| 1. *Area* where more evidence is needed to understand the economic (cost) implications of actions to mitigate and adapt to the health impacts of climate change | Evidence on the health and non-health-related costs and benefits of investing in climate change mitigation and adaptation activities (75.9%) | Unchanged (90%) | Unchanged (80%) |
|  | Evidence on the health and non-health-related costs and benefits of investing in climate change mitigation and adaptation activities (69%) | Unchanged (70%) | Unchanged (80%) |
| 1. Specific sectors where more evidence is needed to understand the economic (cost) implications of actions to mitigate and adapt to the health impacts of climate change | Built environment, building design, healthy homes schemes (60.3%) | Green spaces, green networks, green infrastructure (60%) | Unchanged (60%) |
|  | Healthier diets and sustainability of food supply (48.3%) | Active travel infrastructure and active lifestyles (50%) | Unchanged (50%) |
| 1. Groups or communities where more evidence is needed to understand the economic (cost) implications of actions to mitigate and adapt to the health impacts of climate change | Different income groups (e.g. richer and poorer households) (65.5%) | Unchanged (60%) | Unchanged (80%) |
|  | Different income groups (e.g. richer and poorer households) (50%) | Unchanged (50%) | Unchanged (80%) |

Table 4. Differences in top two research priorities, by population density

| **Survey question** | **Top two research priorities (% listed as one of three top priorities)** | | |
| --- | --- | --- | --- |
|  | **All LAs** | **LAs with 10 lowest population densities** | **LAs with 10 highest population densities** |
| 1. Areas where more evidence is needed by your local authority on ways to engage the public in local action to mitigate and adapt to the health impacts of climate change | The public acceptability of local actions (e.g. low traffic neighbourhoods) (84.5%) | Unchanged (60%) | Unchanged but order reversed (80%) |
|  | Public understandings of climate change and its impacts on people’s health (74.1%) | Unchanged (60%) | Unchanged but order reversed (90%) |
| 1. Groups or communities where more evidence is needed on effective ways to engage the public in local level actions to mitigate and adapt to the health impacts of climate change on health | Different income groups (e.g. richer and poorer households) (65.5%) | Unchanged (80%) | Communities from different ethnic and cultural backgrounds (70%) |
|  | Communities facing barriers to decent housing and local services (44.8%) | Communities from different ethnic and cultural backgrounds (40%) | Different age groups (e.g. children; older people) (50%) |
| 1. *Area* where more evidence is needed to understand the economic (cost) implications of actions to mitigate and adapt to the health impacts of climate change | Evidence on the health and non-health-related costs and benefits of investing in climate change mitigation and adaptation activities (75.9%) | Unchanged (70%) | Unchanged (80%) |
|  | Evidence on the health and non-health-related costs and benefits of investing in climate change mitigation and adaptation activities (69%) | Unchanged (60%) | Unchanged (70%) |
| 1. Specific sectors where more evidence is needed to understand the economic (cost) implications of actions to mitigate and adapt to the health impacts of climate change | Built environment, building design, healthy homes schemes (60.3%) | Unchanged (60%) | Unchanged (60%) |
|  | Healthier diets and sustainability of food supply (48.3%) | Unchanged (40%) | Unchanged (50%) |
| 1. Groups or communities where more evidence is needed to understand the economic (cost) implications of actions to mitigate and adapt to the health impacts of climate change | Different income groups (e.g. richer and poorer households) (65.5%) | Unchanged (80%) | All communities (60%) |
|  | Communities facing barriers to decent housing and local services (50%) | Unchanged (40%) | Different income groups (e.g. richer and poorer households) (65.5%) |

## References

1. Office for National Statistics. Population profiles for local authorities in England 2022 [Available from: <https://www.ons.gov.uk/peoplepopulationandcommunity/populationandmigration/populationestimates/articles/populationprofilesforlocalauthoritiesinengland/2020-12-14>.
