# Supplementary material for: Priorities for research to support local authority action on health and climate change: a study in England
Source: BMC Public Health. 2023 Oct 10;23:1965. doi: 10.1186/s12889-023-16717-1 (PMC10566048; doi:10.1186/s12889-023-16717-1)
Supplement: Supplementary file 5 — Additional file 5. Evidence Review and Inclusion/Exclusion Criteria. [file 12889_2023_16717_MOESM5_ESM.docx]

*Supplementary File 5.*

**Evidence Review Results and Inclusion/Exclusion Criteria**

## Table of Contents

[Review One – Public Acceptability of Local Climate Change Mitigation Actions Public](#_Review_One_–_1)

[Review Two – Understanding of the Impact of Climate Change on Health](#_Review_Two_–)

[Review Three – Cost Effectiveness and Budgetary Implications of Climate Change Mitigation](#_Review_Three_–)

[Included Studies Tables](#_Included_Studies_Tables)

[PRISMA Diagrams](#_PRISMA_Diagrams_1)

[Inclusion/Exclusion Criteria](#_Inclusion/exclusion_criteria)

[References](#_References)

## Review One – Public Acceptability of Local Climate Change Mitigation Actions

*Overview*

110 research studies (based on 117 publications) were found dealing with public acceptability of local action related to climate change (for PRISMA diagram, see **Figure 7**). **Figure 1** demonstrates the broader categories within which these studies have been placed, highlighting the relative abundance of studies looking at the public acceptance of energy-related and recycling action. **Figure 2** shows the location of studies that stated the town or city in which the participants were recruited, across Great Britain and Northern Ireland. An account of included studies can be found in **Table 1**.

**Figure 1. Pie chart showing broader categories of included studies (Other category includes the subcategories Pollution, Planning, Finance, and Policy).**

**
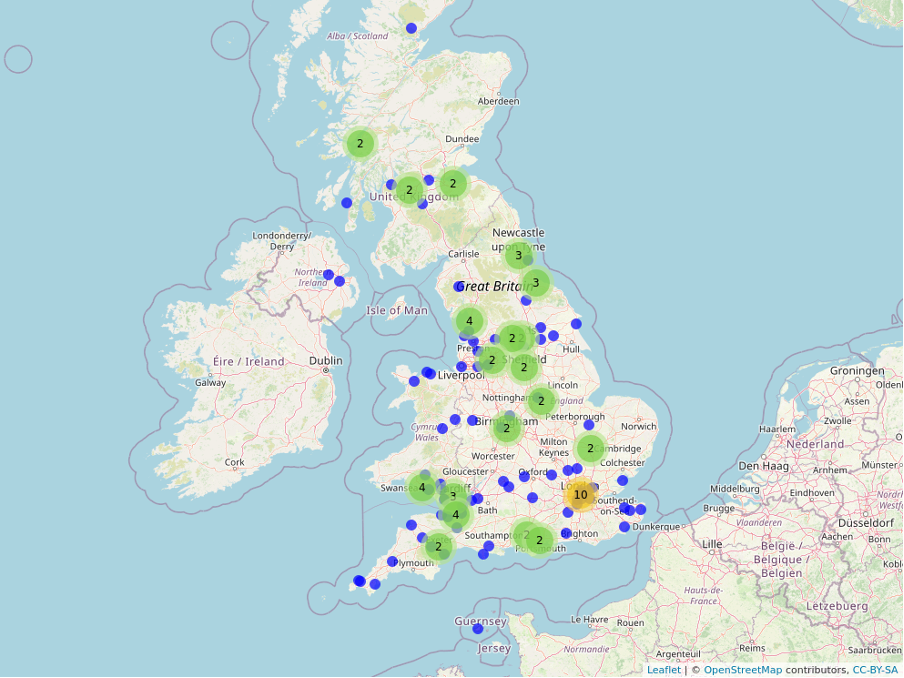
**

**Figure 2. Location of included studies across the UK.**

The majority of studies found looking at the public acceptability of local action that relate in some way to climate change are based on energy sources and renewable energy development siting. **Figure 3** shows the location of these studies in the UK. Studies around wind power and wind turbines were the most common (n=20), followed by bioenergy or biomass (n=4), blue energy (wave or tidal, n=3), and solar power (n=2). Two further studies looked more broadly at renewable energy in their studies. Alternative and fossil fuel sources of energy are also explored for their public acceptance, including around nuclear (n=4), hydrogen (n=3), shale gas extraction (n=6), and coal (n=1).

Further studies, in the area of energy use and infrastructure, relate to the acceptability of high voltage pylons (n=5), local carbon capture and storage (n=8), reduced street lighting (n=1), and energy efficiency schemes in the home (n=1). With only one study each on the latter interventions – reduced street lighting and energy efficiency in the home – and given their potential for health impact, these mark potential gaps of note for the NIHR. In addition, neither engages with the impact of socio-economic status or ethnic group on their findings – the need for which was foregrounded by the survey of Directors of Public Health more generally.


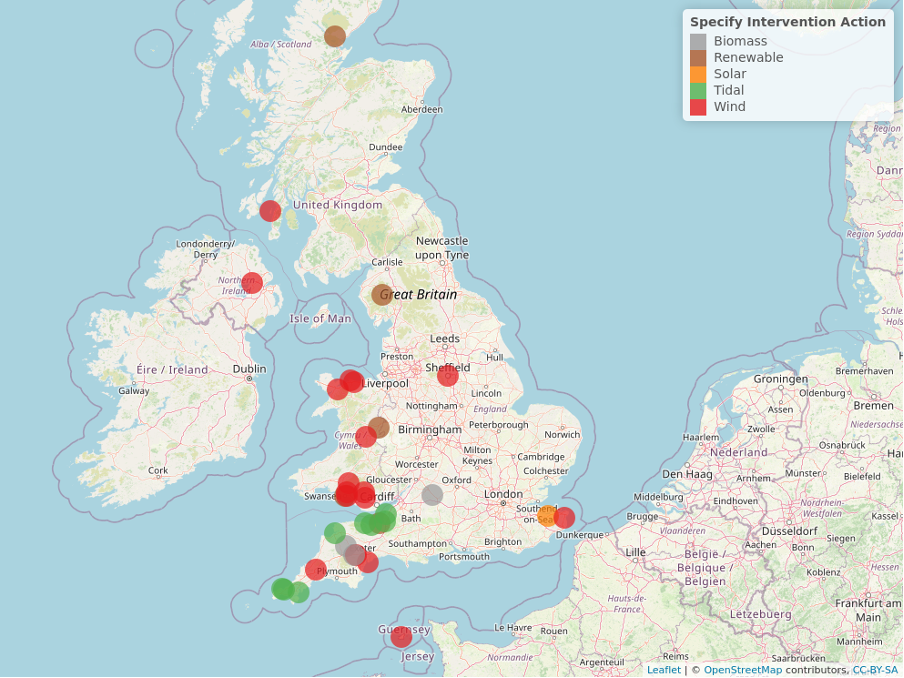


**Figure 3. Map demonstrating locations of energy siting-related studies in the UK.**

A relatively large number of included studies explore existing recycling schemes. These make up the bulk of recycling studies, with 61% of recycling studies covering acceptability of a recycling scheme. Despite this, our other search highlights that there is very little, if any, [economic evaluation](#_Review_Three_–) of recycling schemes. In addition, while there is a relative abundance of studies compared to other interventions, 16 of the 20 included studies (80%) in this area were published between 2000 and 2010, with potential scope for studies to demonstrate more up-to-date analyses. Only seven of these studies included a break down by other variables, such as socio-economic status or ethnic group, demonstrating the need for analysis of how public acceptability of recycling schemes are stratified by other factors.

A number of studies in our search sit in the area between transport and pollution, which may be of direct interest to a variety of NIHR groups. A collection of publications look at the use of air quality information (e.g. uptake, presentation, interpretation, and impact on behaviour), all of which are based on English populations or broader survey data from the UK as a whole (without any meaningful breakdown). There is therefore a gap in studies of air quality information use in Wales, Scotland and Northern Ireland. In addition, none of these studies separate their findings by any socio-demographic or socio-economic variable, indicating another gap in the stratified utilisation of such technologies.

Very few studies were located at the transport-pollution nexus, but those that were included two looking at clean air or low emission zones (covering England and Scotland), two exploring public acceptability of congestion charges (covering England and Scotland), one looking at road pricing (in an English setting), and one more looking at traffic demand management more broadly (in an English setting). There is little coverage, therefore, of Wales and Northern Ireland in the small amount of research available. While the clean air zone research separated its findings by socio-demographic variables (including age, ethnic group, and employment status), and the road pricing studies did so by age, none of the other studies in this area provided a meaningful breakdown that demonstrated the stratification of public acceptability, indicating a clear gap.

Three other interventions within this general theme, with little traction in the research, are also worth highlighting due to their potential relevance to the NIHR. One study exists on vehicle idling, in England, with the intervention being appealing to the self-interest (and therefore shared moral inclinations) of idling drivers. Another is around the public acceptability of speed restrictions in local areas, for which there are two studies (Scotland and Northern Ireland). A final study of interest is around acceptability in relation to banning diesel engines, based in Wales. None of these studies - around idling (or (dis)incentivising the practice), speed restrictions, or the banning of polluting engines - explore how their findings relate to socio-demographic or socio-economic factors. This, and the fact that there are very few studies, indicate significant gaps for potentially relevant areas.

## Review Two – Public Understanding of the Impact of Climate Change on Health

*Overview*

27 research studies (based on 30 publications) – a relatively small amount of research – were found (for PRISMA diagram, see **Figure 8**) based in the UK looking at perceptions of the impact of climate change or a climate-related exposure on health, with the majority of this being undertaken within English settings. An account of included studies can be found in **Table 2**.

The outcome of this search demonstrates the need for more research in the area. Only six studies explore the perceptions of the health impacts of climate change more broadly (see **Figure 4**). Of these, three explore health only as a secondary objective (Parry et al., 2022; Steentjes et al., 2020; Taylor et al., 2019) and two, with more open-ended methodologies, only include health as part of their findings (Darier & Schüle, 1999; Lorenzoni et al., 2006). Both of these latter two studies use data split with other nations than those in the UK. Only one study (Hilary Graham et al., 2019) explores specifically the relationship between climate change and health, though this as part of a contingent valuation methodology, demonstrating a gap of textual and contextual studies looking at perceptions specifically of health impacts. This is particularly the case given that research shows a potentially positive impact of a health framing of climate change across various publics (Dasandi et al., 2022; Myers et al., 2012; Rossa-Roccor et al., 2021), something that may be relevant to local authorities.

**Figure 4. Number of studies researching climate change more broadly and/or specific exposure by health focus within article**

Six studies also looked at public perceptions of heatwaves, half of which treated health as their primary objective, the other half as a secondary objective. Papers looking at flooding (n=7, 26% coverage across all studies) predominantly treated health as a secondary objective (n=7, 26%), with only two papers exploring health as their primary objective. The importance of health in relation to flooding in the perception of the public is clear with one study treating health as part of its findings. Only one study looked at infectious diseases and did so with health as its primary focus. 12 studies (44%) looked at the public’s perception of air pollution as a health concern, with six of these treating health as its primary focus, four as its secondary objective, and two with health as implicit, despite a lack of naming “health” per se. Where climate-related exposures are the key component of the study, more research is needed based on the extent to which the public connect this to climate change.

**Figure 5. Annual number of studies looking at specific exposure or climate change more broadly**

**Figure 5** demonstrates that these studies are not evenly distributed over time. For example, comparing with pre-2010, there have been fewer studies looking at public perceptions of air pollution in relation to health, while there have been a greater number looking at public perceptions of heatwaves and flooding.

In terms of national coverage, England is covered by 16 of the studies (59% of all studies), Wales by three (11%), and Scotland by just one (4%). Northern Ireland had no coverage across the studies. Nine studies (33%) were broader studies covering the UK more generally. This is demonstrated in **Figure 6**. **Figure 7** shows more detail regarding where in those nations, in cases where a specific sample is reported, these studies took place. It is clear from **Figure 6** that there is a lack of research within Scotland, Wales, and Northern Ireland. It is also clear from **Figure 7** that London is overrepresented, while places such as mid-Wales, the Southwest of England, and the Midlands are also poorly represented.

**Figure 6. Coverage of UK nations in studies looking at public perceptions of health and climate change or climate-related exposure.**


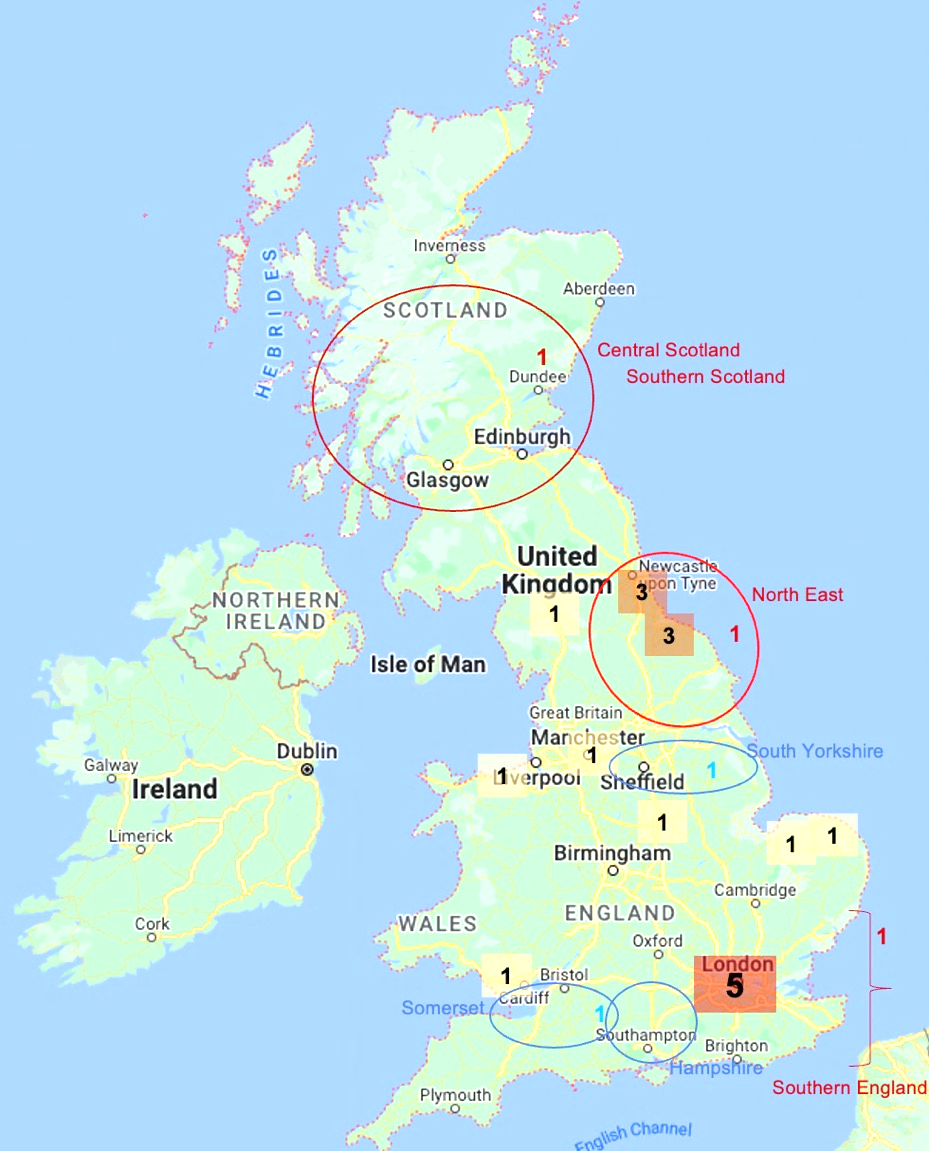


**Figure 7. Map of study locations across the UK (circles indicate broader catchment areas, with blue circles indicating county and red circles indicating a still broader area).**

In terms of methodology, 17 studies used questionnaires (63%), nine studies used interviews (3% of all studies), three used focus groups (11%), and two used contingent valuation methods (7%, e.g. willingness to pay). With the majority of studies using closed question-based methods, there is a clear gap of in-depth qualitative studies to build a meaningful profile of why particular groups are more or less likely to engage with climate change and climate-related exposures as a health issue.

Of those that used interview or focus group data, three studies sampled purposefully across different socio-economic groups or levels of deprivation (Abrahamson et al., 2009; J. Bush et al., 2001; Day, 2007). Further indicators of socio-economic status include: income or financial strain, covered by four studies (15%); educational attainment, also covered by four studies (15%); employment status, covered by one study; and housing tenure, also covered by one study. In all, eight studies (30%) cover socio-economic status in some, by no means homogenous, way. Three studies look specifically at different age groups, meaning that this variable is built into their sample (Abrahamson et al., 2009; Myers et al., 2004; Walkling & Haworth, 2020). Nine other studies separate their findings by age in some way, whether by “generation”, age, or age group. Seven studies separate out their findings by gender. Only one study (Khare et al., 2015) incorporates ethnicity (and this as a binary variable) into its study.

## Review Three – Cost Effectiveness and Budgetary Implications of Climate Change Mitigation

*Overview*

Very little research has explored the short-, medium-, or long-term budgetary implications of climate change mitigation and adaptation or measured the health and non-health costs and benefits of investing in climate change mitigation (see **Table 3**). Given how few relevant studies emerged in the review, two searches were combined. For the PRISMA diagram, see **Figure 10**.

In total only five studies were identified which attempted to quantify either the cost-effectiveness of climate change mitigation actions or their budgetary implications. There was no evidence which measured short-, medium-, or long-term impacts on local authorities, or other local level UK decision-making entities, despite calls for more information from the Directors of Public Health when surveyed.

The five economics studies attempted to quantify either cost-effectiveness or the cost-benefit of a climate mitigation action compared to a do-nothing option. The climate-related exposures within these studies related to waste management, ecology restoration, home energy, air pollution, and transport.

Each of these studies used data from local changes/initiatives, two of which were London-based, but all attempted to generalize findings across the UK narratively and not quantitatively.

All but one study, relating to waste management, included a limited time horizon to quantify comparative costs and outcomes, likely resulting in an inaccurate understanding of the cost-effectiveness of the various climate actions.

All five studies were UK based, with 2/5 conducted in London and the remaining three in Wales, Cambridge and Milton Keynes. Locations seem to be based on what local initiatives exist rather than explicit consideration of the generalisability of the study site. Furthermore, none of the studies explicitly considered the generalisability of the results across other regions. There are therefore significant gaps in the evidence regarding cost-effectiveness and budgetary impacts of climate change mitigation interventions across the UK.

## Included Studies Tables

**Table 1. Public Acceptability Included Studies**

| **Study** | **National context** | **Intervention theme** | **SES variable separated out?** | **Other variable separated out?** |
| --- | --- | --- | --- | --- |
| Aitken (2009) | Scotland | Energy |  |  |
| Alexander et al. (2009) | England | Recycling |  |  |
| Allen et al. (2006) | Scotland | Transport |  |  |
| Allison et al. (2022) | "UK" | Recycling | Income | Gender, household structure |
| Andersson-Hudson et al. (2019) | "UK" | Energy |  |  |
| Bailey et al. (2011) | England | Energy |  |  |
| Barr et al. (2003) | England | Recycling |  |  |
| Batel et al. (2015) | England; Wales | Energy |  |  |
| Batel and Devine‐Wright (2017) | "UK" | Energy |  |  |
| Beaumont et al. (1999) | "UK" | Other: Information |  |  |
| Beebeejaun (2017) | England | Energy |  |  |
| Bees and Williams (2017) | England; Wales | Recycling |  |  |
| Gray et al. (2020) | England | Energy | Housing tenure |  |
| Belton et al. (1994) | Scotland | Recycling | Discusses in relation to employment status | Discusses in relation to age |
| Bench et al. (2005) | England | Recycling |  |  |
| Berry et al. (2011) | Wales | Energy |  |  |
| Bishop and Proctor (1994) | Wales | Energy |  |  |
| Braunholtz (2003) | Scotland | Energy |  |  |
| Broecks et al. (2021) | "UK" | Other: Pollution | Educational attainment | Gender, age |
| Brunsting et al. (2013) | Scotland | Other: Pollution |  |  |
| Judith Bush et al. (2001) | England | Other: Information |  |  |
| CAST (2022) | Wales | Transport |  |  |
| Cotterill et al. (2009) | England | Recycling |  |  |
| Cotton and Devine-Wright (2013) | England | Energy |  |  |
| Davies et al. (2002) | England | Recycling |  |  |
| Davis et al. (2006) | England | Recycling |  |  |
| Davis (2021) | England | Other: Finance |  |  |
| Devine-Wright (2005) | Wales | Energy | Employment status | Gender, age |
| Devine-Wright and Howes (2010) | Wales | Energy |  |  |
| Devine-Wright (2011) | Northern Ireland | Energy |  |  |
| Devine-Wright and Batel (2013) | "UK" | Energy | Education, socio-economic gradient | Gender, age, area of residence, voting intention |
| Devine-Wright (2013) | England | Energy | Education | Gender, age |
| Devine-Wright and Wiersma (2020) | "UK" | Energy | Education, income (representative of island) | Gender, age (representative of island) |
| Dudleston (2000) | Scotland | Energy |  |  |
| Eiser et al. (1988) | England | Energy | Employment status | Gender, age |
| Ellis et al. (2007) | Northern Ireland | Energy |  |  |
| Evans et al. (2011) | England; Scotland | Energy |  |  |
| Evison and Read (2001) | England | Recycling |  |  |
| Gardiner (2012) | "UK" | Energy |  |  |
| Gilbertson et al. (2006) | "UK" | Recycling |  |  |
| Gough et al. (2014) | England | Energy |  | Gender, age (briefly separated) |
| Gough et al. (2018) | England | Energy |  |  |
| Green et al. (2015) | England | Energy |  |  |
| Haddad and de Nazelle (2018) | England; Wales | Energy |  |  |
| Hanley and Nevin (1999) | England | Other: air quality - awareness |  |  |
| Harthorn et al. (2019) | Scotland | Energy |  |  |
| Hinshelwood and McCallum (2001) | England; Wales | Energy |  |  |
| Hooper et al. (2020) | Wales | Energy |  | Gender, age |
| Jesson (2009) | England | Energy |  | Age |
| Jones and Eiser (2009) | England | Recycling |  |  |
| Karousakis and Birol (2008) | England | Energy | Housing tenure, employment status | Gender, age |
| Kurz et al. (2007) | England | Recycling | Education, income |  |
| Lee and Hickman (1989) | Northern Ireland | Recycling | Socio-economic status |  |
| Brook Lyndhurst (2009) | England | Energy |  |  |
| Mabon et al. (2014) | England | Recycling |  |  |
| Mabon et al. (2015) | Scotland | Energy |  |  |
| Martin et al. (2006) | Scotland | Energy |  |  |
| McDonald and Ball (1998) | England | Recycling | Employment status, tax band | Housing type, household structure |
| McDonald et al. (2002) | Scotland | Recycling | Socio-economic status | Age |
| McDonald and Oates (2003) | England | Other: air quality management |  |  |
| McNally et al. (2018) | England | Recycling |  |  |
| Mee and Clewes (2004) | England | Energy | Educational attainment | Age, gender, political orientation |
| Mee and Clewes (2004) | England | Recycling; Other: information, awareness |  |  |
| Mee (2005) | England | Recycling; Other: information, awareness | Employment status, education (degree or not), housing tenure | Ethnic group |
| Morton et al. (2021) | England | Recycling |  |  |
| Nikitas et al. (2018) | Scotland | Transport | Employment status | Sex, age |
| O’Garra and Mourato (2007) | England | Transport |  |  |
| Odioso and Smith (2008) | England | Transport; Energy | Educational attainment, employment status, income | Sex, age, car ownership |
| Parkhill et al. (2014) | England | Transport |  |  |
| Perrin and Barton (2001) | England; Wales | Energy |  |  |
| Perry and Williams (2007) | England | Recycling |  |  |
| Pidgeon, Lorenzoni, et al. (2008) | England | Recycling |  | Gender, age, ethnic group |
| Pidgeon, Henwood, et al. (2008) | England; Wales; Scotland | Energy |  |  |
| Rashid et al. (2021) | England | Energy |  |  |
| Read (1999) | England | Transport |  | Ethnic group |
| Ricci et al. (2006) | England | Recycling |  |  |
| Rispo et al. (2015) | England; Wales | Energy |  |  |
| Robinson and Read (2005) | England | Recycling |  |  |
| Roddis et al. (2018) | England | Recycling |  |  |
| Roddis et al. (2020) | England; Wales; Scotland | Energy |  |  |
| Rogers et al. (2008) | England | Energy |  |  |
| Semwal et al. (2021) | England | Energy |  |  |
| Shammut et al. (2019) | Northern Ireland; Scotland | Transport |  |  |
| Shaw and Maynard (2008) | England | Transport |  |  |
| Shearer et al. (2017) | England | Recycling |  |  |
| Sims and Dent (2007) | England | Recycling |  |  |
| Tatchley et al. (2016) | England | Energy |  |  |
| Thomas et al. (2018) | "UK" | Energy |  | Age |
| Thorpe et al. (2000) | England | Energy |  |  |
| Timlett and Williams (2008) | England | Transport |  |  |
| Tucker and Speirs (2003) | England | Recycling |  |  |
| Turner et al. (2018) | England; Scotland | Recycling |  |  |
| Upham (2009) | Scotland | Transport |  |  |
| Upreti and van der Horst (2004) | England | Energy |  |  |
| Van Alstine and Bastin (2019) | England | Energy |  |  |
| Van de Vyver et al. (2018) | England | Energy |  |  |
| Walker et al. (2014) | England | Transport |  |  |
| Walker et al. (2018) | England | Energy |  |  |
| Warren et al. (2005) | England | Transport; Other: general climate policy |  |  |
| Warren and McFadyen (2010) | Scotland | Energy |  |  |
| West (2004) | Scotland | Energy |  |  |
| Westrom (2020) | England | Energy |  |  |
| Whitmarsh et al. (2015) | Scotland | Energy |  |  |
| Wicki et al. (2022) | England; Wales | Energy | Educational attainment | Gender, age, area type |
| Williams and Kelly (2003) | England | Other: planning |  |  |
| Williams and Cole (2013) | England | Recycling |  | Gender, age |
| Williams et al. (2017) | England | Recycling |  |  |
| Williams et al. (2021) | England | Energy |  |  |
| Wilson and Williams (2007) | Wales | Energy |  |  |

**Table 2. Public Understanding Included Studies**

| **Study** | **National context** | **Health as…** | **SES variable separated out?** | **Other variable separated out?** |
| --- | --- | --- | --- | --- |
| Abrahamson et al. (2009) | England | Primary | Socio-economic status, due to where studies were undertaken (built in) | Age groups (built in) |
| Beaumont et al. (1999) | England; "UK" | Secondary |  |  |
| J. Bush et al. (2001) | England | Primary | Socio-economic status, due to where studies were undertaken (built in) |  |
| Capstick et al. (2013) | Wales | In findings | Housing tenure, employment status | Gender, age |
| Capstick et al. (2015) | England; Wales | Secondary |  |  |
| Carroll et al. (2009) | England | Primary |  |  |
| Darier and Schüle (1999) | England | In findings |  |  |
| Day (2006) | England | Primary | Socio-economic status, due to choice of areas (built in) |  |
| Edgley et al. (2011) | England | Primary | Location (built in) | Age, health status (built in) |
| Erens et al. (2021) | England | Primary | Household type | Age, health |
| H. Graham et al. (2019) | "UK" | Primary | Income band, educational attainment | Gender, age (generation), |
| Graham et al. (2022) | "UK" | Primary |  |  |
| Hodgson and Hitchings (2018) | England | Primary |  |  |
| Howel et al. (2002) | England | Secondary |  | Gender, age, health (chronic illness or not) |
| Istamto et al. (2014) | "UK" | Primary | Educational attainment, financial position (strain), income | Age, gender, health, air pollution concern, policy position |
| Khare et al. (2015) | "UK" | Primary | Location, educational attainment, income | Age group, sex, ethnicity (binary) |
| Lorenzoni et al. (2006) | "UK" | In findings |  | “Representative sample” (built in) |
| Myers et al. (2004) | England | Secondary |  | Age, due to younger sample (built in) |
| Palutikof et al. (2004) | England; Scotland | Secondary |  | Country (i.e. location), age, gender |
| Parry et al. (2022) | England | Secondary |  |  |
| Steentjes et al. (2020) | "UK" | Secondary |  |  |
| Taylor et al. (2019) | "UK" | Secondary |  |  |
| van Wijk et al. (2020) | "UK" | Primary |  | Education (background in natural sciences) |
| Walkling and Haworth (2020) | Wales | Secondary |  | Age (built in) |
| Wall (1973) | England | Implicit |  |  |
| Whitmarsh and Capstick (2018) | England | Secondary | Educational attainment, income | Attainment of vocational science qualification, age, political party, gender |
| Williams and Bird (2003) | England | Implicit |  |  |

**Table 3. Economic Reviews Included Studies**

| **Study** | **National context** | **Exposure** | **Perspective** | **Type of study** |
| --- | --- | --- | --- | --- |
| Craighill and Powell (1996) | England (Milton Keynes) | Waste management | Societal at the LA level | CBA and life cycle assessment: single study comparative assessment |
| Peh et al. (2014) | England (Cambridge) | Ecology restoration | Ecosystem but also includes nature based recreation | CBA: single study comparative assessment |
| Poortinga et al. (2018) | Wales | Housing energy performance investment programme targeting those with low income | Health system; health service for costs but also includes non-health outcomes (food poverty) | CCA and CUA; Single before and after cohort study. ITSA for effects. |
| Peng et al. (2017) | England (London) | Air pollution/air quality using a smart parking system | Individual and city-level | CBA; single study |
| Santos and Fraser (2006) | England (London) | Transport (Central charging scheme) | City-level | CBA: single study |

## PRISMA Diagrams


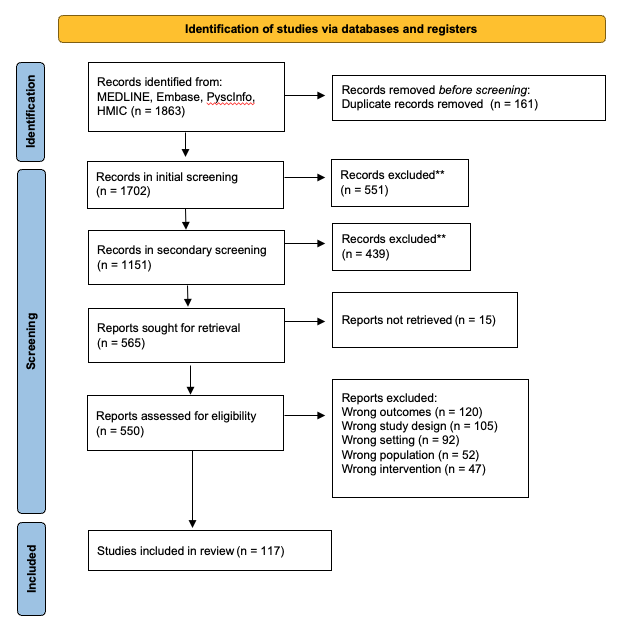


**Figure 8. PRISMA diagram for Review One (Public Acceptability of Local Climate Change Mitigation Action)**

**
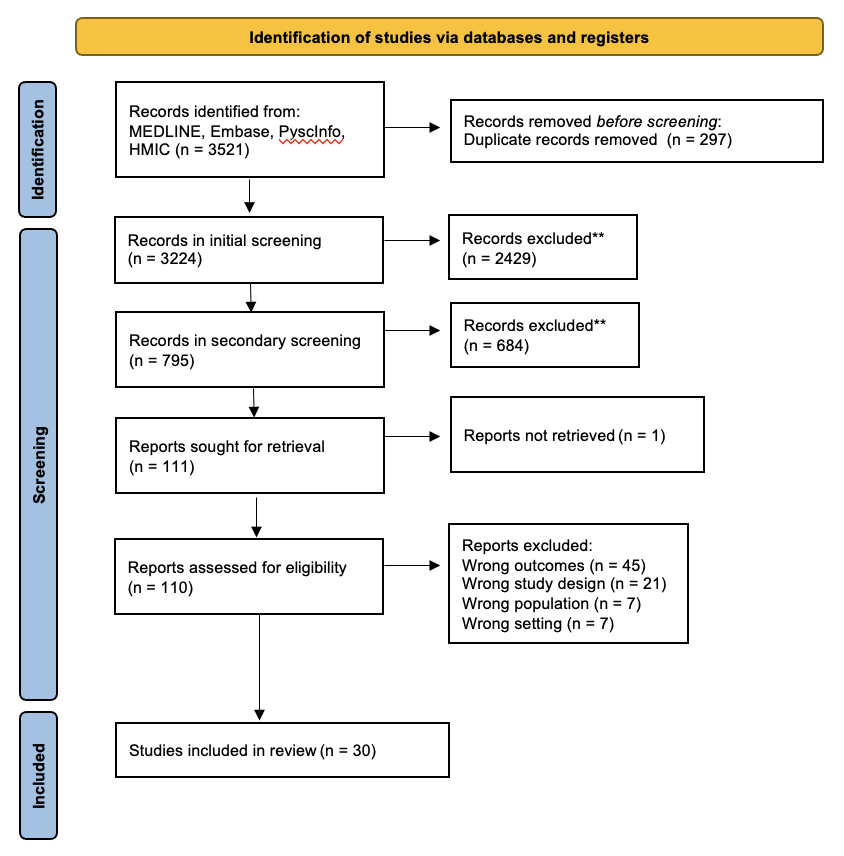
**

**Figure 9. PRISMA diagram for Review Two (Public Understanding of the Health Impacts of Climate Change)**


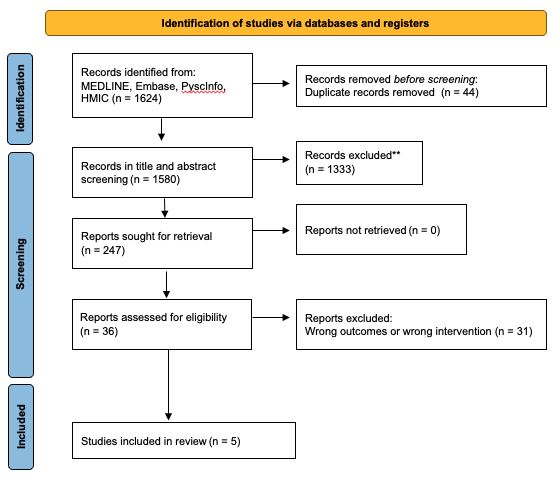


**Figure 10. PRISMA diagram for Review Three (Economic Evaluation)**

## Inclusion/exclusion criteria

### **Review One – Public Acceptability of Local Climate Change Mitigation Actions**

|  | **Inclusion Criteria** | **Exclusion Criteria** |
| --- | --- | --- |
| **S**ample | Any sample of people residing in the UK. No minimum sample size will be implemented. | Any sample of people residing outside of the UK, or predominately outside the UK. |
| **P**henomenon of **I**nterest | Local actions (such as those involving transport, housing, food, green spaces, land and waste management and water supply and quality) to mitigate against climate change or provide adaptation and resilience strategies. | National or international actions to mitigate against climate change or provide adaptation and resilience strategies. |
| **D**esign | Any qualitative or quantitative data collection methods (eg, surveys, questionnaires, interviews, observations, or focus groups) independent of the analysis conducted. | Discussion papers and descriptive papers will be excluded. |
| **E**valuation | Any information on public attitudes or public acceptability of local actions. Such information may be the primary or secondary focus of the study. | Any studies which do not measure or evaluate public acceptability. |
| **R**esearch type | Qualitative (such as interviews or focus groups, social media analysis), quantitative (such as surveys or questionnaires with fixed responses) or mixed methods (such as research which collates a combination of fixed and open-ended responses). Content analyses of social media-based research. | Non-research |

The search string for this review included terms within three facets:

- Climate change
- Public acceptability
- Local government

The search also incorporated, where possible, the National Institute for Health and Care Excellence’s (NICE) UK study filter. Further information for the search strategy can be found [here](https://www.researchregistry.com/browse-the-registry#registryofsystematicreviewsmeta-analyses/registryofsystematicreviewsmeta-analysesdetails/63871d24702cc600230bd1b5/).

Databases searched were OVID Medline(R) and Epub Ahead of Print, In-Process, In-Data-Review & Other Non-Indexed Citations and Daily, Embase, APA PsycINFO, and HMIC Health Management.

### **Review Two – Public understandings of climate change and its impact on people’s health**

|  | **Inclusion Criteria** | **Exclusion Criteria** |
| --- | --- | --- |
| **S**ample | Any sample of people residing in the UK. No minimum sample size will be implemented. | Any sample of people residing outside of the UK, or predominately outside the UK. |
| **P**henomenon of **I**nterest | Public understandings of climate change and its impact on health. | Understandings of those with climate-related expertise regarding the health impacts of climate change |
| **D**esign | Any data collection methods (eg, surveys, questionnaires, interviews, observations, or focus groups) independent of the analysis conducted. | Discussion papers and descriptive papers will be excluded. |
| **E**valuation | Any information on public understandings of the health impacts of climate change. Such information may be the primary or secondary focus of the study | Any studies which do not measure public understandings of the health impacts of climate change. |
| **R**esearch type | Qualitative (such as interviews or focus groups, social media analysis), quantitative (such as surveys or questionnaires with fixed responses) or mixed methods (such as research which collates a combination of fixed and open-ended responses). Content analyses of social media-based research. | Non-research |

The search string for this review included terms within three facets:

- Climate change
- Public understanding
- Local government

The search also incorporated, where possible, the National Institute for Health and Care Excellence’s (NICE) UK study filter. Further information for the search strategy can be found [here](https://www.researchregistry.com/browse-the-registry#registryofsystematicreviewsmeta-analyses/registryofsystematicreviewsmeta-analysesdetails/638717a2b01cee0021d9c1b4/).

Databases searched were OVID Medline(R) and Epub Ahead of Print, In-Process, In-Data-Review & Other Non-Indexed Citations and Daily, Embase, APA PsycINFO, and HMIC Health Management.

### **Review Three – Cost Effectiveness and Budgetary Implications of Climate Change Mitigation**

|  | **Inclusion Criteria** | **Exclusion Criteria** |
| --- | --- | --- |
| **S**ample | UK study | Non-UK study |
| **P**henomenon of **I**nterest | Research evidence measuring or evaluating the costs and benefits (however defined and including health and non-health outcomes) of interventions to mitigate against climate change or research looking at the financial (across all sectors) implications of interventions to mitigate against climate change | Research evidence measuring or evaluating the costs and benefits or research looking at the financial (across all sectors) implications of interventions unrelated to climate change mitigation and adaptation |
| **D**esign | Cost-effectiveness analysis, Cost-utility analysis, Cost-benefit analysis, Cost-minimisation, Budget impact analyses, SROI, ROI, business case forecasting. | Discussion papers and descriptive papers will be excluded. |
| **E**valuation | Any studies looking at either costs and benefits or financial implications. Such information may be the primary or secondary focus of the study. | Any studies not looking at either costs and benefits or financial implications |
| **R**esearch type | Original research. | Non-research |

The search string for this review included terms within three facets:

- Climate change and Net Zero policy
- Economic terms
- Local (within the concept of “local action”)

The search also incorporated, where possible, the National Institute for Health and Care Excellence’s (NICE) UK study filter. Further information for the search strategy can be found [here](https://www.researchregistry.com/browse-the-registry#registryofsystematicreviewsmeta-analyses/registryofsystematicreviewsmeta-analysesdetails/63888b7552bc5100257aee3c/).

Databases searched were OVID Medline(R) and Epub Ahead of Print, In-Process, In-Data-Review & Other Non-Indexed Citations and Daily, Embase, APA PsycINFO, HMIC Health Management, IDEAS, and econLit.

## References

Abrahamson, V., Wolf, J., Lorenzoni, I., Fenn, B., Kovats, S., Wilkinson, P., Adger, W. N., & Raine, R. (2009). Perceptions of heatwave risks to health: interview-based study of older people in London and Norwich, UK. *Journal of Public Health*, *31*(1), 119-126. <https://ovidsp.ovid.com/ovidweb.cgi?T=JS&CSC=Y&NEWS=N&PAGE=fulltext&D=med7&AN=19052099>

Aitken, M. (2009). Wind Power Planning Controversies and the Construction of ‘Expert’ and ‘Lay’ Knowledges. *Science as Culture*, *18*(1), 47-64. <https://doi.org/10.1080/09505430802385682>

Alexander, C., Smaje, C., Timlett, R., & Williams, I. (2009). Improving social technologies for recycling. *Proceedings of the Institution of Civil Engineers - Waste and Resource Management*, *162*(1), 15-28. <https://doi.org/10.1680/warm.2009.162.1.15>

Allen, S., Gaunt, M., & Rye, T. (2006). An investigation into the reasons for the rejection of congestion charging by the citizens of Edinburgh.

Allison, A. L., Lorencatto, F., Michie, S., & Miodownik, M. (2022). Barriers and Enablers to Food Waste Recycling: A Mixed Methods Study amongst UK Citizens. *International Journal of Environmental Research and Public Health*, *19(5) (no pagination)*. <https://doi.org/https://dx.doi.org/10.3390/ijerph19052729>

Andersson-Hudson, J., Rose, J., Humphrey, M., Knight, W., & O'Hara, S. (2019). The structure of attitudes towards shale gas extraction in the United Kingdom. *Energy Policy*, *129*, 693-697. <https://doi.org/https://doi.org/10.1016/j.enpol.2019.02.056>

Bailey, I., West, J., & Whitehead, I. (2011). Out of Sight but Not out of Mind? Public Perceptions of Wave Energy. *Journal of Environmental Policy & Planning*, *13*(2), 139-157. <https://doi.org/10.1080/1523908X.2011.573632>

Barr, S., Ford, N. J., & Gilg, A. W. (2003). Attitudes towards Recycling Household Waste in Exeter, Devon: Quantitative and qualitative approaches. *Local Environment*, *8*(4), 407-421. <https://doi.org/10.1080/13549830306667>

Batel, S., Devine-Wright, P., Wold, L., Egeland, H., Jacobsen, G., & Aas, O. (2015). The role of (de-)essentialisation within siting conflicts: An interdisciplinary approach. *Journal of Environmental Psychology*, *44*, 149-159. <https://doi.org/https://dx.doi.org/10.1016/j.jenvp.2015.10.004>

Batel, S., & Devine‐Wright, P. (2017). Energy colonialism and the role of the global in local responses to new energy infrastructures in the UK: A critical and exploratory empirical analysis. *Antipode*, *49*(1), 3-22.

Beaumont, R., Hamilton, R. S., Machin, N., Perks, J., & Williams, I. D. (1999). Social awareness of air quality information. *Science of the Total Environment*, *235(1-3)*, 319-329. <https://ovidsp.ovid.com/ovidweb.cgi?T=JS&CSC=Y&NEWS=N&PAGE=fulltext&D=emed6&AN=29565270>

Beebeejaun, Y. (2017). Exploring the intersections between local knowledge and environmental regulation: A study of shale gas extraction in Texas and Lancashire. *Environment and Planning C: Politics and Space*, *35*(3), 417-433. <https://doi.org/10.1177/0263774x16664905>

Bees, A. D., & Williams, I. D. (2017). Explaining the differences in household food waste collection and treatment provisions between local authorities in England and Wales. *Waste Management*, *70*, 222-235. <https://ovidsp.ovid.com/ovidweb.cgi?T=JS&CSC=Y&NEWS=N&PAGE=fulltext&D=med14&AN=28918870>

Belton, V., Crowe, D. V., Matthews, R., & Scott, S. (1994). A survey of public attitudes to recycling in Glasgow (U.K.). *Waste Management and Research*, *12(4)*, 351-367. <https://doi.org/https://dx.doi.org/10.1006/wmre.1994.1024>

Bench, M. L., Woodard, R., Harder, M. K., & Stantzos, N. (2005). Waste minimisation: Home digestion trials of biodegradable waste. *Resources, Conservation and Recycling*, *45*(1), 84-94. <https://doi.org/https://doi.org/10.1016/j.resconrec.2005.02.003>

Berry, R., Higgs, G., Fry, R., & Langford, M. (2011). Web-based GIS Approaches to Enhance Public Participation in Wind Farm Planning [<https://doi.org/10.1111/j.1467-9671.2011.01240.x>]. *Transactions in GIS*, *15*(2), 147-172. <https://doi.org/https://doi.org/10.1111/j.1467-9671.2011.01240.x>

Bishop, K., & Proctor, A. (1994). *Love them or loathe them?: public attitudes towards wind farms in Wales*. Department of City and Regional Planning, University of Wales Cardiff.

Braunholtz, S. (2003). Public Attitudes to Windfarms: A Survey of Local Residents inScotland. <Http://www.scotland.gov.uk/library5/environment/pawslr.pdf>, Scottish ExecutiveSocial Research/MORI Scotland.

Broecks, K., Jack, C., Ter Mors, E., Boomsma, C., & Shackley, S. (2021). How do people perceive carbon capture and storage for industrial processes? Examining factors underlying public opinion in the Netherlands and the United Kingdom. *Energy Research & Social Science*, *81*, 102236.

Brook Lyndhurst. (2009). Enhancing participation in kitchen waste collections. *Defra Waste & Resources Evidence Programme (WR0209), Final Project Report*.

Brunsting, S., Pol, M., Mastop, J., Kaiser, M., Zimmer, R., Shackley, S., Mabon, L., Howell, R., Hepplewhite, F., Loveridge, R., Mazurowski, M., & Rybicki, C. (2013). Social Site Characterisation for CO2 Storage Operations to Inform Public Engagement in Poland and Scotland. *Energy Procedia*, *37*, 7327-7336. <https://doi.org/https://doi.org/10.1016/j.egypro.2013.06.671>

Bush, J., Moffatt, S., & Dunn, C. (2001). 'Even the birds round here cough': stigma, air pollution and health in Teesside. *Health & Place*, *7*(1), 47-56. <https://ovidsp.ovid.com/ovidweb.cgi?T=JS&CSC=Y&NEWS=N&PAGE=fulltext&D=med4&AN=11165155>

Bush, J., Moffatt, S., & Dunn, C. E. (2001). Keeping the public informed? Public negotiation of air quality information. *Public Understanding of Science*, *10*(2), 213-229. <https://doi.org/10.3109/a036866>

Capstick, S. B., Demski, C. C., Sposato, R. G., Pidgeon, N. F., Spence, A., & Corner, A. J. (2015). Public perception of climate change in Britain following the winter 2013/2014 flooding.

Capstick, S. B., Pidgeon, N., & Whitehead, M. (2013). Public perceptions of climate change in Wales: Summary findings of a survey of the Welsh public conducted during November and December 2012.

Carroll, B., Morbey, H., Balogh, R., & Araoz, G. (2009). Flooded homes, broken bonds, the meaning of home, psychological processes and their impact on psychological health in a disaster. *Health & Place*, *15*(2), 540-547.

CAST. (2022). *CAST BRIEFING 15: Travel attitudes and behaviours in Cardiff 2021: Results from the first wave of the Cardiff Travel Survey conducted in May-July 2021* (CAST BRIEFINGS, Issue. <https://cast.ac.uk/wp-content/uploads/2022/09/Briefing-15.pdf>

Cotterill, S., John, P., Liu, H., & Nomura, H. (2009). Mobilizing citizen effort to enhance environmental outcomes: A randomized controlled trial of a door-to-door recycling campaign. *Journal of Environmental Management*, *91*(2), 403-410. <https://doi.org/https://doi.org/10.1016/j.jenvman.2009.09.010>

Cotton, M., & Devine-Wright, P. (2013). Putting pylons into place: a UK case study of public perspectives on the impacts of high voltage overhead transmission lines. *Journal of Environmental Planning and Management*, *56*(8), 1225-1245. <https://doi.org/10.1080/09640568.2012.716756>

Craighill, A. L., & Powell, J. C. (1996). Lifecycle assessment and economic evaluation of recycling: A case study. *Resources, Conservation and Recycling*, *17*(2), 75-96. <https://doi.org/https://doi.org/10.1016/0921-3449(96)01105-6>

Darier, E., & Schüle, R. (1999). Think globally, act locally'? Climate change and public participation in Manchester and Frankfurt. *Local environment*, *4*(3), 317-329.

Dasandi, N., Graham, H., Hudson, D., Jankin, S., vanHeerde-Hudson, J., & Watts, N. (2022). Positive, global, and health or environment framing bolsters public support for climate policies. *Communications Earth & Environment*, *3*(1), 239. <https://doi.org/10.1038/s43247-022-00571-x>

Davies, J., Foxall, G. R., & Pallister, J. (2002). Beyond the Intention–Behaviour Mythology: An Integrated Model of Recycling. *Marketing Theory*, *2*(1), 29-113. <https://doi.org/10.1177/1470593102002001645>

Davis, G., Phillips, P. S., Read, A. D., & Iida, Y. (2006). Demonstrating the need for the development of internal research capacity: Understanding recycling participation using the Theory of Planned Behaviour in West Oxfordshire, UK. *Resources, Conservation and Recycling*, *46(2)*, 115-127. <https://doi.org/https://dx.doi.org/10.1016/j.resconrec.2005.07.001>

Davis, M. (2021). Community Municipal Investments: Accelerating the Potential of Local Net Zero Strategies.

Day, R. (2007). Place and the experience of air quality. *Health & Place*, *13*(1), 249-260. <https://ovidsp.ovid.com/ovidweb.cgi?T=JS&CSC=Y&NEWS=N&PAGE=fulltext&D=med6&AN=16500135>

Day, R. J. (2006). Traffic-related air pollution and perceived health risk: Lay assessment of an everyday hazard. *Health, Risk & Society*, *8*(3), 305-322. <https://ovidsp.ovid.com/ovidweb.cgi?T=JS&CSC=Y&NEWS=N&PAGE=fulltext&D=psyc5&AN=2006-13210-007>

Devine-Wright, P. (2005). Local aspects of UK renewable energy development: exploring public beliefs and policy implications. *Local Environment*, *10*(1), 57-69. <https://doi.org/10.1080/1354983042000309315>

Devine-Wright, P. (2011). Enhancing local distinctiveness fosters public acceptance of tidal energy: A UK case study. *Energy Policy*, *39*(1), 83-93. <https://doi.org/https://doi.org/10.1016/j.enpol.2010.09.012>

Devine-Wright, P. (2013). Explaining “NIMBY” Objections to a Power Line:The Role of Personal, Place Attachment and Project-Related Factors. *Environment and Behavior*, *45*(6), 761-781. <https://doi.org/10.1177/0013916512440435>

Devine-Wright, P., & Batel, S. (2013). Explaining public preferences for high voltage pylon designs: An empirical study of perceived fit in a rural landscape. *Land Use Policy*, *31*, 640-649. <https://doi.org/https://doi.org/10.1016/j.landusepol.2012.09.011>

Devine-Wright, P., & Howes, Y. (2010). Disruption to place attachment and the protection of restorative environments: A wind energy case study. *Journal of Environmental Psychology*, *30*(3), 271-280. <https://doi.org/https://dx.doi.org/10.1016/j.jenvp.2010.01.008>

Devine-Wright, P., & Wiersma, B. (2020). Understanding community acceptance of a potential offshore wind energy project in different locations: An island-based analysis of ‘place-technology fit’. *Energy Policy*, *137*, 111086. <https://doi.org/https://doi.org/10.1016/j.enpol.2019.111086>

Dudleston, A. (2000). Public attitudes towards wind farms in Scotland: results of a residents survey.

Edgley, A., Pilnick, A., & Clarke, M. (2011). 'The air still wasn't good ... everywhere I went I was surrounded': Lay perceptions of air quality and health. *Health Sociology Review*, *20*(1), 97-108. <https://ovidsp.ovid.com/ovidweb.cgi?T=JS&CSC=Y&NEWS=N&PAGE=fulltext&D=psyc8&AN=2011-11002-008>

Eiser, J., Spears, R., & Webley, P. (1988). Predicting attitudes to oil and to nuclear energy. *Journal of Environmental Psychology*, *8*(2), 141-147. <https://doi.org/https://dx.doi.org/10.1016/S0272-4944%2888%2980004-5>

Ellis, G., Barry, J., & Robinson, C. (2007). Many ways to say ‘no’, different ways to say ‘yes’: Applying Q-Methodology to understand public acceptance of wind farm proposals. *Journal of Environmental Planning and Management*, *50*(4), 517-551. <https://doi.org/10.1080/09640560701402075>

Erens, B., Williams, L., Exley, J., Ettelt, S., Manacorda, T., Hajat, S., & Mays, N. (2021). Public attitudes to, and behaviours taken during, hot weather by vulnerable groups: results from a national survey in England. *BMC Public Health*, *21*(1), 1631. <https://ovidsp.ovid.com/ovidweb.cgi?T=JS&CSC=Y&NEWS=N&PAGE=fulltext&D=med19&AN=34488695>

Evans, B., Parks, J., & Theobald, K. (2011). Urban wind power and the private sector: community benefits, social acceptance and public engagement. *Journal of Environmental Planning and Management*, *54*(2), 227-244. <https://doi.org/10.1080/09640568.2010.505829>

Evison, T., & Read, A. D. (2001). Local Authority recycling and waste — awareness publicity/promotion. *Resources, Conservation and Recycling*, *32*(3), 275-291. <https://doi.org/https://doi.org/10.1016/S0921-3449(01)00066-0>

Gardiner, B. (2012). *Wind farms: For or against?* <https://yougov.co.uk/topics/politics/articles-reports/2012/04/03/wind-farms-and-renewable-energy>

Gilbertson, J., Stevens, M., Stiell, B., Thorogood, N., & Warm Front Study, G. (2006). Home is where the hearth is: grant recipients' views of England's home energy efficiency scheme (Warm Front). *Soc Sci Med*, *63*(4), 946-956. <https://ovidsp.ovid.com/ovidweb.cgi?T=JS&CSC=Y&NEWS=N&PAGE=fulltext&D=med6&AN=16616807http://openurl.york.ac.uk/openurl/44YORK/44YORK_services_page?sid=OVID:medline&id=pmid:16616807&id=doi:10.1016%2Fj.socscimed.2006.02.021&issn=0277-9536&isbn=&volume=63&i>

Gough, C., Cunningham, R., & Mander, S. (2018). Understanding key elements in establishing a social license for CCS: An empirical approach. *International Journal of Greenhouse Gas Control*, *68*, 16-25. <https://doi.org/https://doi.org/10.1016/j.ijggc.2017.11.003>

Gough, C., O׳Keefe, L., & Mander, S. (2014). Public perceptions of CO2 transportation in pipelines. *Energy Policy*, *70*, 106-114. <https://doi.org/https://doi.org/10.1016/j.enpol.2014.03.039>

Graham, H., de Bell, S., Hanley, N., Jarvis, S., & White, P. C. (2019). Willingness to pay for policies to reduce future deaths from climate change: evidence from a British survey. *public health*, *174*, 110-117.

Graham, H., Harrison, A., & Lampard, P. (2022). Public Perceptions of Climate Change and Its Health Impacts: Taking Account of People's Exposure to Floods and Air Pollution. *International Journal of Environmental Research and Public Health*, *19(4) (no pagination)*. <https://ovidsp.ovid.com/ovidweb.cgi?T=JS&CSC=Y&NEWS=N&PAGE=fulltext&D=emexa&AN=2015677415>

Graham, H., White, P., Cotton, J., & McManus, S. (2019). Flood- and Weather-Damaged Homes and Mental Health: An Analysis Using England's Mental Health Survey. *International Journal of Environmental Research & Public Health [Electronic Resource]*, *16*(18), 05. <https://ovidsp.ovid.com/ovidweb.cgi?T=JS&CSC=Y&NEWS=N&PAGE=fulltext&D=med16&AN=31491859>

Gray, D., Snodin, H., & Bullen, A. (2020). *Exploring the evidence on potential issues associatedwith trialling hydrogen heating in communities: A Literature Review and Focus Group Study.*

Green, J., Perkins, C., Steinbach, R., & Edwards, P. (2015). Reduced street lighting at night and health: A rapid appraisal of public views in England and Wales. *Health and Place*, *34*, 171-180. <https://doi.org/https://dx.doi.org/10.1016/j.healthplace.2015.05.011>

Haddad, H., & de Nazelle, A. (2018). The role of personal air pollution sensors and smartphone technology in changing travel behaviour. *Journal of Transport & Health*, *11*, 230-243. <https://doi.org/https://dx.doi.org/10.1016/j.jth.2018.08.001>

Hanley, N., & Nevin, C. (1999). Appraising renewable energy developments in remote communities: the case of the North Assynt Estate, Scotland. *Energy Policy*, *27*(9), 527-547. <https://doi.org/https://doi.org/10.1016/S0301-4215(99)00023-3>

Harthorn, B. H., Halcomb, L., Partridge, T., Thomas, M., Enders, C., & Pidgeon, N. (2019). Health risk perception and shale development in the UK and US. *Health, Risk & Society*, *21*(1-2), 35-56. <https://doi.org/10.1080/13698575.2019.1601685>

Hinshelwood, E., & McCallum, D. (2001). Examining approaches to renewables consultation. Lessons from Awel Aman Tawe community wind farm project.

Hodgson, A., & Hitchings, R. (2018). Urban air pollution perception through the experience of social practices: Talking about breathing with recreational runners in London. *Health & Place*, *53*, 26-33. <https://ovidsp.ovid.com/ovidweb.cgi?T=JS&CSC=Y&NEWS=N&PAGE=fulltext&D=psyc15&AN=2018-47047-005>

Hooper, T., Hattam, C., Edwards-Jones, A., & Beaumont, N. (2020). Public perceptions of tidal energy: Can you predict social acceptability across coastal communities in England? *Marine Policy*, *119*, 104057. <https://doi.org/https://doi.org/10.1016/j.marpol.2020.104057>

Howel, D., Moffatt, S., Prince, H., Bush, J., & Dunn, C. E. (2002). Urban air quality in North-East England: Exploring the influences on local views and perceptions. *Risk Analysis*, *22(1)*, 121-130. <https://ovidsp.ovid.com/ovidweb.cgi?T=JS&CSC=Y&NEWS=N&PAGE=fulltext&D=emed7&AN=34258739>

Istamto, T., Houthuijs, D., & Lebret, E. (2014). Willingness to pay to avoid health risks from road-traffic-related air pollution and noise across five countries. *Science of the Total Environment*, *497-498*, 420-429. <https://ovidsp.ovid.com/ovidweb.cgi?T=JS&CSC=Y&NEWS=N&PAGE=fulltext&D=emed15&AN=600013963>

Jesson, J. (2009). Household waste recycling behavior: A market segmentation model. *Social Marketing Quarterly*, *15*(2), 25-38. <https://doi.org/https://dx.doi.org/10.1080/15245000902957326>

Karousakis, K., & Birol, E. (2008). Investigating household preferences for kerbside recycling services in London: A choice experiment approach. *Journal of Environmental Management*, *88*(4), 1099-1108. <https://doi.org/https://doi.org/10.1016/j.jenvman.2007.05.015>

Khare, S., Hajat, S., Kovats, S., Lefevre, C. E., de Bruin, W. B., Dessai, S., & Bone, A. (2015). Heat protection behaviour in the UK: results of an online survey after the 2013 heatwave. *BMC Public Health*, *15*, 878. <https://ovidsp.ovid.com/ovidweb.cgi?T=JS&CSC=Y&NEWS=N&PAGE=fulltext&D=med12&AN=26357923>

Kurz, T., Linden, M., & Sheehy, N. (2007). Attitudinal and community influences on participation in new curbside recycling initiatives in Northern Ireland. *Environment and Behavior*, *39*(3), 367-391. <https://doi.org/https://dx.doi.org/10.1177/0013916506294152>

Lorenzoni, I., Leiserowitz, A., De Franca Doria, M., Poortinga, W., & Pidgeon, N. F. (2006). Cross-National Comparisons of Image Associations with "Global Warming" and "Climate Change" Among Laypeople in the United States of America and Great Britain. *Journal of Risk Research*, *9*(3), 265-281. <https://ovidsp.ovid.com/ovidweb.cgi?T=JS&CSC=Y&NEWS=N&PAGE=fulltext&D=psyc5&AN=2006-06158-006>

Mabon, L., Shackley, S., Blackford, J. C., Stahl, H., & Miller, A. (2015). Local perceptions of the QICS experimental offshore CO2 release: Results from social science research. *International Journal of Greenhouse Gas Control*, *38*, 18-25. <https://doi.org/https://doi.org/10.1016/j.ijggc.2014.10.022>

Mabon, L., Shackley, S., & Bower-Bir, N. (2014). Perceptions of sub-seabed carbon dioxide storage in Scotland and implications for policy: A qualitative study. *Marine Policy*, *45*, 9-15. <https://doi.org/https://doi.org/10.1016/j.marpol.2013.11.011>

Martin, M., Williams, I. D., & Clark, M. (2006). Social, cultural and structural influences on household waste recycling: A case study. *Resources, Conservation and Recycling*, *48*(4), 357-395. <https://doi.org/https://doi.org/10.1016/j.resconrec.2005.09.005>

McDonald, J. S., Hession, M., Rickard, A., Nieuwenhuijsen, M. J., & Kendall, M. (2002). Air quality management in UK local authorities: Public understanding and participation. *Journal of Environmental Planning and Management*, *45*(4), 571-590. <https://doi.org/10.1080/09640560220143567>

McDonald, S., & Ball, R. (1998). Public participation in plastics recycling schemes. *Resources, Conservation and Recycling*, *22*(3), 123-141. <https://doi.org/https://doi.org/10.1016/S0921-3449(97)00044-X>

McDonald, S., & Oates, C. (2003). Reasons for non-participation in a kerbside recycling scheme. *Resources, Conservation and Recycling*, *39*(4), 369-385. <https://doi.org/https://doi.org/10.1016/S0921-3449(03)00020-X>

McNally, H., Howley, P., & Cotton, M. (2018). Public perceptions of shale gas in the UK: framing effects and decision heuristics. *Energy, Ecology and Environment*, *3*(6), 305-316. <https://doi.org/10.1007/s40974-018-0102-2>

Mee, N. (2005). A Communications Strategy for Kerbside Recycling. *Journal of Marketing Communications*, *11*(4), 297-308. <https://doi.org/https://dx.doi.org/10.1080/13527260500124265>

Mee, N., & Clewes, D. (2004). The influence of corporate communications on recycling behaviour. *Corporate Communications*, *9*(4), 265-275. <https://doi.org/https://dx.doi.org/10.1108/13563280410571004>

Morton, C., Mattioli, G., & Anable, J. (2021). Public acceptability towards Low Emission Zones: The role of attitudes, norms, emotions, and trust. *Transportation Research Part A: Policy and Practice*, *150*, 256-270. <https://doi.org/https://doi.org/10.1016/j.tra.2021.06.007>

Myers, G., Boyes, E., & Stanisstreet, M. (2004). School students' ideas about air pollution: Knowledge and attitudes. *Research in Science & Technological Education*, *22*(2), 133-152. <https://ovidsp.ovid.com/ovidweb.cgi?T=JS&CSC=Y&NEWS=N&PAGE=fulltext&D=psyc4&AN=2004-21982-001>

Myers, T. A., Nisbet, M. C., Maibach, E. W., & Leiserowitz, A. A. (2012). A public health frame arouses hopeful emotions about climate change. *Climatic Change*, *113*(3), 1105-1112.

Nikitas, A., Avineri, E., & Parkhurst, G. (2018). Understanding the public acceptability of road pricing and the roles of older age, social norms, pro-social values and trust for urban policy-making: The case of Bristol. *Cities*, *79*, 78-91. <https://doi.org/https://doi.org/10.1016/j.cities.2018.02.024>

O’Garra, T., & Mourato, S. (2007). Public Preferences for Hydrogen Buses: Comparing Interval Data, OLS and Quantile Regression Approaches. *Environmental and Resource Economics*, *36*(4), 389-411. <https://doi.org/10.1007/s10640-006-9024-0>

Odioso, M. S., & Smith, M. C. (2008, 25-25 April 2008). Perceptions of congestion charging: Lessons for U.S. cities from London and Stockholm. 2008 IEEE Systems and Information Engineering Design Symposium,

Palutikof, J. P., Agnew, M. D., & Hoar, M. R. (2004). Public perceptions of unusually warm weather in the UK: impacts, responses and adaptations. *Climate Research*, *26*(1), 43-59.

Parkhill, K. A., Butler, C., & Pidgeon, N. F. (2014). Landscapes of Threat? Exploring Discourses of Stigma around Large Energy Developments. *Landscape Research*, *39*(5), 566-582. <https://doi.org/10.1080/01426397.2013.775232>

Parry, S., McCarthy, S. R., & Clark, J. (2022). Young people's engagement with climate change issues through digital media - a content analysis. *Child & Adolescent Mental Health*, *27*(1), 30-38. <https://ovidsp.ovid.com/ovidweb.cgi?T=JS&CSC=Y&NEWS=N&PAGE=fulltext&D=med20&AN=34904349>

Peh, K. S. H., Balmford, A., Field, R. H., Lamb, A., Birch, J. C., Bradbury, R. B., Brown, C., Butchart, S. H. M., Lester, M., Morrison, R., Sedgwick, I., Soans, C., Stattersfield, A. J., Stroh, P. A., Swetnam, R. D., Thomas, D. H. L., Walpole, M., Warrington, S., & Hughes, F. M. R. (2014). Benefits and costs of ecological restoration: Rapid assessment of changing ecosystem service values at a U.K. wetland [<https://doi.org/10.1002/ece3.1248>]. *Ecology and Evolution*, *4*(20), 3875-3886. <https://doi.org/https://doi.org/10.1002/ece3.1248>

Peng, G. C. A., Nunes, M. B., & Zheng, L. (2017). Impacts of low citizen awareness and usage in smart city services: the case of London’s smart parking system. *Information Systems and e-Business Management*, *15*, 845-876.

Perrin, D., & Barton, J. (2001). Issues associated with transforming household attitudes and opinions into materials recovery: a review of two kerbside recycling schemes. *Resources, Conservation and Recycling*, *33*(1), 61-74. <https://doi.org/https://doi.org/10.1016/S0921-3449(01)00075-1>

Perry, G. D. R., & Williams, I. D. (2007). The participation of ethnic minorities in kerbside recycling: A case study. *Resources, Conservation and Recycling*, *49*(3), 308-323. <https://doi.org/https://doi.org/10.1016/j.resconrec.2006.02.006>

Pidgeon, N. F., Henwood, K., Parkhill, K. A., Venables, D., & Simmons, P. (2008). Living with nuclear power in Britain: A mixed-methods study.

Pidgeon, N. F., Lorenzoni, I., & Poortinga, W. (2008). Climate change or nuclear power—No thanks! A quantitative study of public perceptions and risk framing in Britain. *Global Environmental Change*, *18*(1), 69-85. <https://doi.org/https://doi.org/10.1016/j.gloenvcha.2007.09.005>

Poortinga, W., Rodgers, S. E., Lyons, R. A., Anderson, P., Tweed, C., Grey, C., Jiang, S., Johnson, R., Watkins, A., & Winfield, T. G. (2018). The health impacts of energy performance investments in low-income areas: a mixed-methods approach. *Public Health Research*, *6*(5), 1-182.

Rashid, R., Chong, F., Islam, S., Bryant, M., & McEachan, R. R. C. (2021). Taking a deep breath: a qualitative study exploring acceptability and perceived unintended consequences of charging clean air zones and air quality improvement initiatives amongst low-income, multi-ethnic communities in Bradford, UK. *BMC Public Health*, *21(1)*, 1305. <https://ovidsp.ovid.com/ovidweb.cgi?T=JS&CSC=Y&NEWS=N&PAGE=fulltext&D=emed22&AN=635527970>

Read, A. D. (1999). 'A weekly doorstep recycling collection, I had no idea we could!': Overcoming the local barriers to participation. *Resources, Conservation and Recycling*, *26(3-4)*, 217-249. <https://doi.org/https://dx.doi.org/10.1016/S0921-3449%2899%2900008-7>

Ricci, M., Flynn, R., & Bellaby, P. (2006). Public Attitudes towards Hydrogen energy: Preliminary analysis of findings from focus groups in London, Teesside and Wales. *University of Salford*.

Rispo, A., Williams, I. D., & Shaw, P. J. (2015). Source segregation and food waste prevention activities in high-density households in a deprived urban area. *Waste Management*, *44*, 15-27. <https://doi.org/https://dx.doi.org/10.1016/j.wasman.2015.04.010>

Robinson, G. M., & Read, A. D. (2005). Recycling behaviour in a London Borough: Results from large-scale household surveys. *Resources, Conservation and Recycling*, *45*(1), 70-83. <https://doi.org/https://doi.org/10.1016/j.resconrec.2005.02.002>

Roddis, P., Carver, S., Dallimer, M., Norman, P., & Ziv, G. (2018). The role of community acceptance in planning outcomes for onshore wind and solar farms: An energy justice analysis. *Applied Energy*, *226*, 353-364. <https://doi.org/https://doi.org/10.1016/j.apenergy.2018.05.087>

Roddis, P., Roelich, K., Tran, K., Carver, S., Dallimer, M., & Ziv, G. (2020). What shapes community acceptance of large-scale solar farms? A case study of the UK’s first ‘nationally significant’solar farm. *Solar Energy*, *209*, 235-244.

Rogers, J. C., Simmons, E. A., Convery, I., & Weatherall, A. (2008). Public perceptions of opportunities for community-based renewable energy projects. *Energy Policy*, *36*(11), 4217-4226. <https://doi.org/https://doi.org/10.1016/j.enpol.2008.07.028>

Rossa-Roccor, V., Giang, A., & Kershaw, P. (2021). Framing climate change as a human health issue: enough to tip the scale in climate policy? *The Lancet Planetary Health*, *5*(8), e553-e559.

Santos, G., & Fraser, G. (2006). Road pricing: lessons from London. *Economic Policy*, *21*(46), 264-310.

Semwal, T., Milton, K., Jepson, R., & Kelly, M. P. (2021). Tweeting about twenty: an analysis of interest, public sentiments and opinion about 20mph speed restrictions in two UK cities. *BMC public health*, *21(1)*, 2016. <https://doi.org/https://dx.doi.org/10.1186/s12889-021-12084-x>

Shammut, M., Cao, M., Zhang, Y., Papaix, C., Liu, Y., & Gao, X. (2019). Banning Diesel Vehicles in London: Is 2040 Too Late? *Energies*, *12*(18), 3495. <https://www.mdpi.com/1996-1073/12/18/3495>

Shaw, P. J., & Maynard, S. J. (2008). The potential of financial incentives to enhance householders’ kerbside recycling behaviour. *Waste Management*, *28*(10), 1732-1741. <https://doi.org/https://doi.org/10.1016/j.wasman.2007.08.008>

Shearer, L., Gatersleben, B., Morse, S., Smyth, M., & Hunt, S. (2017). A problem unstuck? Evaluating the effectiveness of sticker prompts for encouraging household food waste recycling behaviour. *Waste Management*, *60*, 164-172. <https://doi.org/https://doi.org/10.1016/j.wasman.2016.09.036>

Sims, S., & Dent, P. (2007). Property stigma: wind farms are just the latest fashion. *Journal of Property Investment & Finance*, *25*(6), 626-651. <https://doi.org/10.1108/14635780710829315>

Steentjes, K., Demski, C., Seabrook, A., Corner, A., & Pidgeon, N. (2020). British public perceptions of climate risk, adaptation options and resilience (RESiL RISK): topline findings of a GB survey conducted in October 2019.

Tatchley, C., Paton, H., Robertson, E., Minderman, J., Hanley, N., & Park, K. (2016). Drivers of Public Attitudes towards Small Wind Turbines in the UK. *PLOS ONE*, *11*(3), e0152033. <https://doi.org/10.1371/journal.pone.0152033>

Taylor, A., Dessai, S., & Bruine de Bruin, W. (2019). Public priorities and expectations of climate change impacts in the United Kingdom. *Journal of Risk Research*, *22*(2), 150-160. <https://ovidsp.ovid.com/ovidweb.cgi?T=JS&CSC=Y&NEWS=N&PAGE=fulltext&D=psyc16&AN=2019-18263-002>

Thomas, G., Pidgeon, N., & Roberts, E. (2018). Ambivalence, naturalness and normality in public perceptions of carbon capture and storage in biomass, fossil energy, and industrial applications in the United Kingdom. *Energy Research & Social Science*, *46*, 1-9. <https://doi.org/https://doi.org/10.1016/j.erss.2018.06.007>

Thorpe, N., Hills, P., & Jaensirisak, S. (2000). Public attitudes to TDM measures: a comparative study. *Transport Policy*, *7*(4), 243-257. <https://doi.org/https://doi.org/10.1016/S0967-070X(00)00007-X>

Timlett, R. E., & Williams, I. D. (2008). Public participation and recycling performance in England: A comparison of tools for behaviour change. *Resources, Conservation and Recycling*, *52*(4), 622-634. <https://doi.org/https://doi.org/10.1016/j.resconrec.2007.08.003>

Tucker, P., & Speirs, D. (2003). Attitudes and behavioural change in household waste management behaviours. *Journal of environmental planning and management*, *46*(2), 289-307.

Turner, K., Jepson, R., MacDonald, B., Kelly, P., Biggs, H., & Baker, G. (2018). Developing and refining a programme theory for understanding how twenty mile per hour speed limits impact health. *Journal of Transport & Health*, *10*, 92-110. <https://doi.org/https://dx.doi.org/10.1016/j.jth.2018.08.004>

Upham, P. (2009). Applying environmental-behaviour concepts to renewable energy siting controversy: Reflections on a longitudinal bioenergy case study. *Energy Policy*, *37*(11), 4273-4283. <https://doi.org/https://doi.org/10.1016/j.enpol.2009.05.027>

Upreti, B. R., & van der Horst, D. (2004). National renewable energy policy and local opposition in the UK: the failed development of a biomass electricity plant. *Biomass and Bioenergy*, *26*(1), 61-69. <https://doi.org/https://doi.org/10.1016/S0961-9534(03)00099-0>

Van Alstine, J., & Bastin, C. (2019). Establishing the UK Hydrogen Corridor: Socio-Economic, Environmental & Regulatory Issues.

Van de Vyver, J., Abrams, D., Hopthrow, T., Purewal, K., de Moura, G. R., & Meleady, R. (2018). Motivating the selfish to stop idling: Self-interest cues can improve environmentally relevant driver behaviour. *Transportation Research Part F: Traffic Psychology and Behaviour*, *54*, 79-85. <https://doi.org/https://doi.org/10.1016/j.trf.2018.01.015>

van Wijk, M., Naing, S., Diaz Franchy, S., Heslop, R. T., Novoa Lozano, I., Vila, J., & Balleste-Delpierre, C. (2020). Perception and knowledge of the effect of climate change on infectious diseases within the general public: A multinational cross-sectional survey-based study. *PLoS ONE [Electronic Resource]*, *15*(11), e0241579. <https://ovidsp.ovid.com/ovidweb.cgi?T=JS&CSC=Y&NEWS=N&PAGE=fulltext&D=med18&AN=33151991>

Walker, B. J. A., Kurz, T., & Russel, D. (2018). Towards an understanding of when non-climate frames can generate public support for climate change policy. *Environment and Behavior*, *50*(7), 781-806. <https://doi.org/10.1177/0013916517713299>

Walker, B. J. A., Wiersma, B., & Bailey, E. (2014). Community benefits, framing and the social acceptance of offshore wind farms: an experimental study in England. *Energy Research & Social Science*, *3*, 46-54.

Walkling, B., & Haworth, B. T. (2020). Flood risk perceptions and coping capacities among the retired population, with implications for risk communication: A study of residents in a north Wales coastal town, UK. *International Journal of Disaster Risk Reduction*, *51*, 101793. <https://ovidsp.ovid.com/ovidweb.cgi?T=JS&CSC=Y&NEWS=N&PAGE=fulltext&D=pmnm5&AN=32834976>

Wall, G. (1973). Public response to air pollution in South Yorkshire, England. *Environment and Behavior*, *5*(2), 219-248. <https://ovidsp.ovid.com/ovidweb.cgi?T=JS&CSC=Y&NEWS=N&PAGE=fulltext&D=psyc2&AN=1974-05024-001>

Warren, C. R., Lumsden, C., O'Dowd, S., & Birnie, R. V. (2005). ‘Green On Green’: Public perceptions of wind power in Scotland and Ireland. *Journal of Environmental Planning and Management*, *48*(6), 853-875. <https://doi.org/10.1080/09640560500294376>

Warren, C. R., & McFadyen, M. (2010). Does community ownership affect public attitudes to wind energy? A case study from south-west Scotland. *Land Use Policy*, *27*(2), 204-213. <https://doi.org/https://doi.org/10.1016/j.landusepol.2008.12.010>

West, M. S. R. I. f. R. S. (2004). *Attitudes Towards Renewable Energy in Devon. (July 8, 2006)*. <http://www.regensw.co.uk/content-download/DevonMORIPollReport091104.pdf>

Westrom, M. (2020). Winds of change: Legitimacy, withdrawal, and interdependency from a decentralized wind-to-hydrogen regime in Orkney, Scotland. *Energy Research & Social Science*, *60*, 101332. <https://doi.org/https://doi.org/10.1016/j.erss.2019.101332>

Whitmarsh, L., & Capstick, S. (2018). Perceptions of climate change. *Clayton, Susan [Ed]; Manning, Christie [Ed] (2018) Psychology and climate change: Human perceptions, impacts, and responses (pp 13-33) xii, 299 pp San Diego, CA, US: Elsevier Academic Press; US*, 13-33. <https://ovidsp.ovid.com/ovidweb.cgi?T=JS&CSC=Y&NEWS=N&PAGE=fulltext&D=psyc15&AN=2018-00751-002>

Whitmarsh, L., Nash, N., Upham, P., Lloyd, A., Verdon, J. P., & Kendall, J. M. (2015). UK public perceptions of shale gas hydraulic fracturing: The role of audience, message and contextual factors on risk perceptions and policy support. *Applied Energy*, *160*, 419-430. <https://doi.org/https://doi.org/10.1016/j.apenergy.2015.09.004>

Wicki, M., Hofer, K., & Kaufmann, D. (2022). Planning instruments enhance the acceptance of urban densification. *Proc Natl Acad Sci U S A*, *119*(38), e2201780119. <https://doi.org/https://dx.doi.org/10.1073/pnas.2201780119>

Williams, I. D., & Bird, A. (2003). Public perceptions of air quality and quality of life in urban and suburban areas of London. *Journal of Environmental Monitoring*, *5(2)*, 253-259. <https://ovidsp.ovid.com/ovidweb.cgi?T=JS&CSC=Y&NEWS=N&PAGE=fulltext&D=emed8&AN=36469514>

Williams, I. D., & Cole, C. (2013). The impact of alternate weekly collections on waste arisings. *Science of The Total Environment*, *445-446*, 29-40. <https://doi.org/https://doi.org/10.1016/j.scitotenv.2012.12.024>

Williams, I. D., & Kelly, J. (2003). Green waste collection and the public's recycling behaviour in the Borough of Wyre, England. *Resources, Conservation and Recycling*, *38*(2), 139-159. <https://doi.org/https://doi.org/10.1016/S0921-3449(02)00106-4>

Williams, L., Macnaghten, P., Davies, R., & Curtis, S. (2017). Framing ‘fracking’: Exploring public perceptions of hydraulic fracturing in the United Kingdom. *Public Understanding of Science*, *26*(1), 89-104. <https://doi.org/10.1177/0963662515595159>

Williams, R., Jack, C., Gamboa, D., & Shackley, S. (2021). Decarbonising steel production using CO2 Capture and Storage (CCS): Results of focus group discussions in a Welsh steel-making community. *International Journal of Greenhouse Gas Control*, *104*, 103218. <https://doi.org/https://doi.org/10.1016/j.ijggc.2020.103218>

Wilson, C. D. H., & Williams, I. D. (2007). Kerbside collection: A case study from the north-west of England. *Resources, Conservation and Recycling*, *52*(2), 381-394. <https://doi.org/https://doi.org/10.1016/j.resconrec.2007.02.006>
